# Workflow for Fine-Tuning and Evaluating DNA Language Models for Specific Genomics Issues

**DOI:** 10.21769/BioProtoc.5676

**Published:** 2026-04-20

**Authors:** Kazuki Nakamae, Hidemasa Bono

**Affiliations:** 1 PtBio Inc., Higashi-Hiroshima, Japan; 2 Genome Editing Innovation Center, Hiroshima University, Higashi-Hiroshima, Japan; 3 Graduate School of Integrated Sciences for Life, Hiroshima University, Higashi-Hiroshima, Japan; 4 Database Center for Life Science, Joint Support-Center for Data Science Research, Research Organization of Information and Systems, Wakashiba, Kashiwa, Japan

**Keywords:** DNABERT-2, DNA language model, Cytosine base editor, RNA off-target, Promoter, EPDnew, Fine-tuning, Deep learning

## Abstract

DNA language models, such as DNABERT-2, have recently enabled the accurate prediction of functional sequence elements across species. However, the practical, protocol-style steps needed to transform these resources into training datasets, fine-tune the official DNABERT-2 model, and evaluate classifier performance have not been explicitly described. Herein, we present a step-by-step computational protocol for preparing training data, fine-tuning DNABERT-2, and evaluating sequence-level binary classifiers using readily available command-line tools. The protocol has been demonstrated using RNA off-target sites induced by cytosine base editors, detected by our PiCTURE pipeline from RNA sequencing (RNA-seq) data, and extended to core promoter prediction using the EPDnew database. We describe how to derive positive and negative sequence sets into DNABERT-2 compatible datasets, and fine-tune the official pretrained model of DNABERT-2 using the datasets. We also demonstrate how to compute the standard performance metrics and compare the model outputs with the baselines. This protocol will help researchers adapt DNA foundation models to new genomic tasks, including the safety assessment of genome editing tools and the functional annotation of regulatory sequences.

Key features

• A step-by-step pipeline has been provided for preparing labeled DNA sequence datasets for DNABERT-2 fine-tuning.

• The reproducibility of the official DNABERT-2 implementation and scripts for binary classification is shown.

• Application of PiCTURE-derived C-to-U (T) substitution sites for off-target RNA risk prediction.

• An extension to core promoter prediction using promoter datasets from EPDnew is described.

**This protocol is used in:** International Journal of Molecular Sciences (2025), DOI: 10.3390/ijms26041723

## Graphical overview


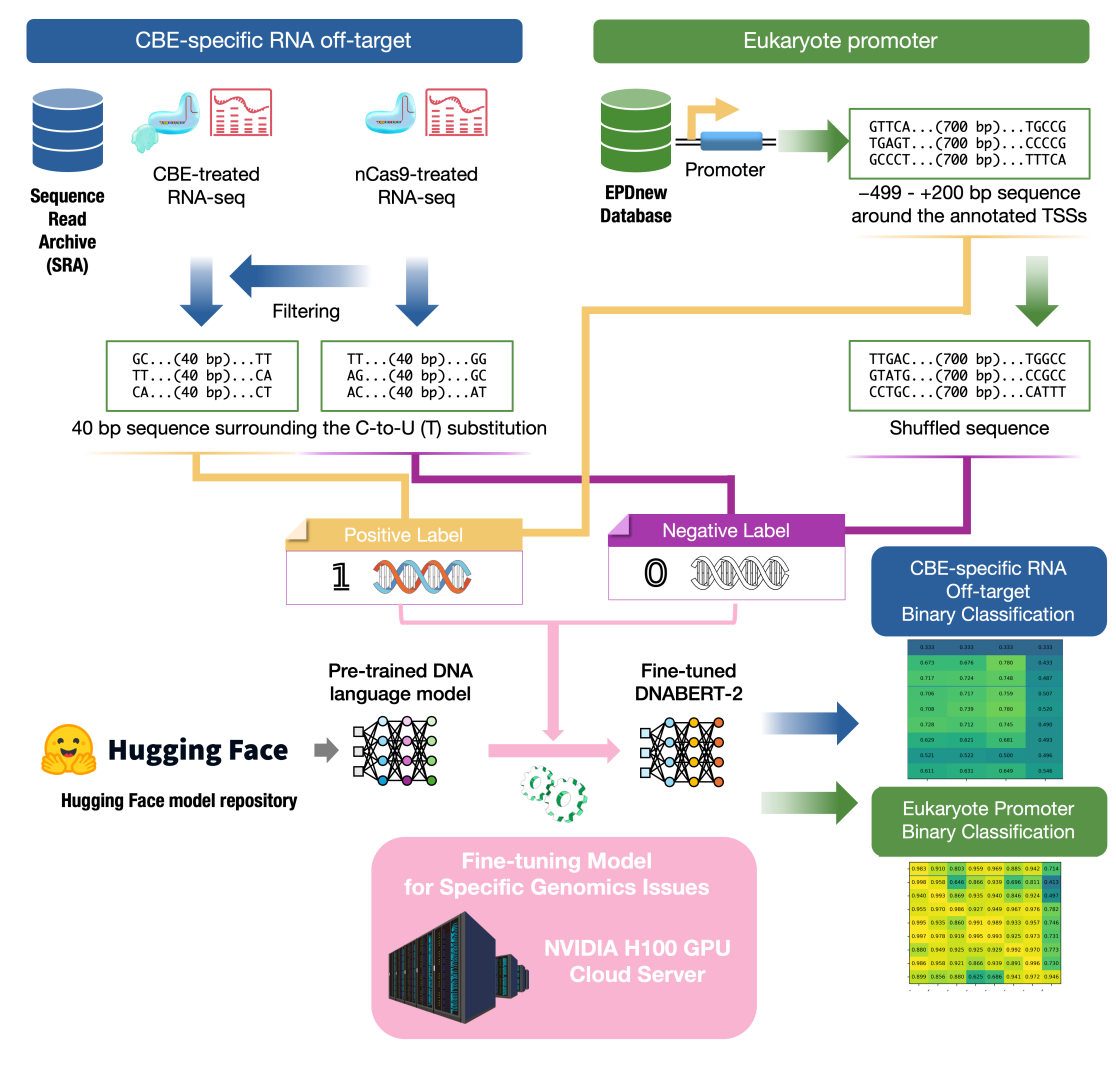


**Graphical overview of the fine-tuning pipeline for predicting cytosine base editors (CBE)-specific RNA off-targets and eukaryote promoters using DNABERT-2.** Images of the DNA and RNA-seq were obtained from TogoTV (© 2016 DBCLS TogoTV, CC-BY-4.0 https://creativecommons.org/licenses/by/4.0/).

## Background

DNA language models trained on large-scale genomic corpora have emerged as powerful tools for the prediction of functional sequence elements [1–6]. DNABERT-2 is a foundation model pretrained on data comprising multi-species genomes using byte-pair encoding tokenization and an efficient transformer architecture [6]. It has demonstrated state-of-the-art performance in diverse genome-understanding tasks.

Cytosine base editors (CBEs) introduce C-to-U (T) substitutions without generating double-strand breaks [7–9]; however, some base editors can also cause unintended C-to-U (T) substitutions on RNA, called RNA off-target [10–12]. RNA off-target sequences are often enriched in canonical ACW motifs (W = A or T/U) [10]; however, non-canonical events are also observed and are more difficult to characterize [13,14]. To systematically profile such off-targets, we previously developed PiCTURE (Pipeline for CRISPR-induced Transcriptome-wide Unintended RNA Editing) (https://github.com/KazukiNakamae/PiCTURE) [15], which processes RNA-seq data from CBE-transfected samples and outputs high-confidence C-to-U (T) substitution sites and their local sequence contexts [13].

In our recent study, PiCTURE outputs were used to fine-tune DNABERT-2 into a binary classifier, the SNL (RNAoffScan v2) model (https://github.com/KazukiNakamae/RNAOffScan), which distinguishes CBE-induced RNA substitutions from background substitution sites and outperforms motif-only baselines in terms of accuracy, precision, recall, and F1 score [13]. Hence, DNA foundation models can capture both canonical and noncanonical sequence signals associated with off-target editing.

In addition to genome editing safety, DNABERT-2 can be adapted to a broad range of regulatory genomics tasks [6]. In this protocol, eukaryotic promoter databases, such as EPDnew [16], provide experimentally validated transcription start sites (TSSs) and flanking promoter sequences for multiple species. These resources enable the systematic construction of a positive dataset for promoter prediction and allow the evaluation of model performance [17,18].

However, the practical steps required to construct training datasets from PiCTURE outputs or the EPDnew database, fine-tune the official DNABERT-2 implementation, and evaluate the classifier performance have not been described in a protocol format. The following protocol provides a reproducible, end-to-end workflow for (i) generating labeled DNA sequence datasets from PiCTURE outputs and the EPDnew database, (ii) formatting them for DNABERT-2, (iii) fine-tuning DNABERT-2 using the official pretrained model, and (iv) evaluating the performance relative to motif-based or other machine learning-based baselines. By following this protocol, researchers can adapt DNABERT-2 to new sequence-level classification tasks, quantify model performance, and leverage DNA language models for genome-editing risk assessment and eukaryotic regulatory genomics.

## Equipment

*Note: This protocol is entirely computational and does not include wet-lab experiments.*

1. MacBook Pro (14 in, 2024), Apple M4 Max 16 cores, LPDDR5 RAM 128 GB Hynix, SSD 2 TB, MacOS version 26.0 (Apple Inc.)

*Note: Unless otherwise specified, the analysis in this protocol was performed using a MAC device. Most analysis commands are compatible with a Linux PC.*

2. Linux Server, Intel Xeon w7-3445, 20 cores, RAM 1024 GB, SSD (system) 3.8 TB M.2 NVMe × 2, GPU RTX 4500 Ada (VRAM: 24 GB), Ubuntu version 22.04.5 LTS (NABE International, Inc.)

3. Sakura's Cloud Server, Intel(R) Xeon(R) Platinum 8480+, 24 cores, 240 GB RAM, 2 TB SSD, NVIDIA H100 (VRAM: 80 GB), Ubuntu version 22.04.5 LTS (SAKURA Internet Inc.)

*Note: The NVIDIA H100 (80 GB VRAM) was primarily used for fine-tuning DNABERT-2. We also confirmed that the workflow can be executed on five NVIDIA Tesla V100 SXM2 GPUs (16 GB VRAM each) using distributed/sharded training. Note that this setup provides 80 GB of aggregate device memory rather than a single 80 GB memory pool. In our environment, the workflow typically completes within one day on an H100, whereas it requires approximately 3–7 days on the multi-V100 setup, depending on the distributed-training configuration and input length.*

## Software and datasets

All software and dataset information are provided in Table 1.

**Table 1.** Software tools and datasets used in this pipeline

Type	Software/dataset/resource	Version (SHA256 hash, Git commit hash, Zenodo DOI, Manifest digest, MD5sum hash)	Date	License	Access (free or paid)
Software	DNABERT-2 model and fine-tuning scripts (MAGICS-LAB/DNABERT_2) [6]	Main branch (Git commit hash: d87d29f5570f6d8238de734c932697ef657be59a)	Accessed on 2025-12-09	Apache-2.0	Free via GitHub website (https://github.com/MAGICS-LAB/DNABERT_2)
Software	PyTorch [19]	Version 2.4.1+cu121-cp38-cp38-linux_x86_64 (SHA256 hash: cb4f502f910b47e1e366ccf7b231dac2967d2efb47d4b8cb33fc63b4bc5eeed8)	Accessed on 2025-12	BSD 3-Clause License	Free via PyTorch website (https://pytorch.org/)
Software	Transformers [20]	Version 4.29.2 (Git commit hash: ba7054533fa455e8b2dd35feb077e0c7aae646b3)	Accessed on 2025-12	Apache 2.0 License	Free via GitHub website (https://github.com/huggingface/transformers)
Software	An unofficial collection of helper scripts for DNABERT-2 (KazukiNakamae/DNABERT_2_helper)	Version 1.0.0 (DOI: 10.5281/zenodo.18954363)	Accessed on 2026-01-11	MIT License	Free via GitHub website (https://github.com/KazukiNakamae/DNABERT_2_helper)
Software	PiCTURE pipeline for RNA off-target detection (KazukiNakamae/PiCTURE) [13]	Version 1.0.0 (DOI: 10.5281/zenodo.18954750)	Accessed on 2025-12-10	MIT License	Free via GitHub website (https://github.com/KazukiNakamae/PiCTURE)
Software	A collection of scripts for data processing of plants’ promoters (KazukiNakamae/plant_promoter_classifier)	Version 1.0.0 (DOI: 10.5281/zenodo.18963418)	Accessed on 2025-12-10	MIT License	Free via GitHub website (https://github.com/KazukiNakamae/plant_promoter_classifier)
Software	A collection of scripts for data processing of mammals’ promoters (KazukiNakamae/mammal_promoter_classifier)	Version 1.0.0 (DOI: 10.5281/zenodo.18963493)	Accessed on 2025-12-10	MIT License	Free via GitHub website (https://github.com/KazukiNakamae/mammal_promoter_classifier)
Software	A collection of scripts for data processing of mammals’ ncRNA promoters (KazukiNakamae/mammal_ncRNA_promoter_classifier)	Version 1.0.0 (DOI: 10.5281/zenodo.18963677)	Accessed on 2025-12-10	MIT License	Free via GitHub website (https://github.com/KazukiNakamae/mammal_ncRNA_promoter_classifier)
Software	A collection of scripts for data processing of birds’ promoters (KazukiNakamae/bird_promoter_classifier)	Version 1.0.0 (DOI: 10.5281/zenodo.18963776)	Accessed on 2025-12-10	MIT License	Free via GitHub website (https://github.com/KazukiNakamae/bird_promoter_classifier)
Software	A collection of scripts for data processing of insects’ promoters (KazukiNakamae/insect_promoter_classifier)	Version 1.0.0 (DOI: 10.5281/zenodo.18963825)	Accessed on 2025-12-10	MIT License	Free via GitHub website (https://github.com/KazukiNakamae/insect_promoter_classifier)
Software	A collection of scripts for data processing of fish’s promoters (KazukiNakamae/fish_promoter_classifier)	Version 1.0.0 (DOI: 10.5281/zenodo.18963878)	Accessed on 2025-12-10	MIT License	Free via GitHub website (https://github.com/KazukiNakamae/fish_promoter_classifier)
Software	A collection of scripts for data processing of nematoda’s promoters (KazukiNakamae/nematoda_promoter_classifier)	Version 1.0.0 (DOI: 10.5281/zenodo.18963993)	Accessed on 2025-12-10	MIT License	Free via GitHub website (https://github.com/KazukiNakamae/nematoda_promoter_classifier)
Software	A collection of scripts for data processing of yeasts’ promoters (KazukiNakamae/yeast_promoter_classifier)	Version 1.0.0 (DOI: 10.5281/zenodo.18964048)	Accessed on 2025-12-10	MIT License	Free via GitHub website (https://github.com/KazukiNakamae/yeast_promoter_classifier)
Software	A script for di-nucleotide shuffling (KazukiNakamae/shuffle_fasta_di)	Version 1.0.0 (DOI: 10.5281/zenodo.18964299)	Accessed on 2026-03-10	MIT License	Free via GitHub website (https://github.com/KazukiNakamae/shuffle_fasta_di)
Software	Trim Galore (Docker container used in PiCTURE) [21]	Version 0.6.7 (SHA256 hash: ffc30fbdb4d28bd385465f661ed717168d10f77640371d237167d521f57b62d2)	Accessed on 2025-12-10	GNU General Public License v3.0	Free via Docker Hub website (https://hub.docker.com/r/clinicalgenomics/trim_galore)
Software	GATK (Docker container used in PiCTURE) [22,23]	Version 4.4.0.0 (SHA256 hash: e7996ba655225c1cde0a1faec6a113e217758310af2cf99b00d61dae8ec6e9f2)	Accessed on 2025-12-10	BSD 3-Clause License	Free via Docker Hub website (https://hub.docker.com/r/broadinstitute/gatk)
Software	BCFtools (Docker container used in PiCTURE) [24]	Version 1.16 (SHA256 hash: 8b358095bc71232c407d72e5b8d88d727dbff39aedf928a76aca6d6bc8e35e60)	Accessed on 2025-12-10	MIT/Expat License	Free via Docker Hub website (https://hub.docker.com/r/staphb/bcftools)
Software	Picard (Docker container used in PiCTURE) [25]	Version 2.18.1 (SHA256 hash: 7a7cc0d02da5f1e0e487d47559a510f2ae318a8727e39e0f95a46a59656d0c25)	Accessed on 2025-12-10	MIT License	Free via Docker Hub website (https://hub.docker.com/r/mgibio/picard-cwl)
Software	MultiQC (Docker container used in PiCTURE) [26]	Version 1.14 (SHA256 hash: ca2e4b4bed61e484a301b3ee585efb73abc68c46a6944ec3735942c9e3017193)	Accessed on 2025-12-10	GNU General Public License v3.0	Free via Docker Hub website (https://hub.docker.com/r/ewels/multiqc)
Software	pysam helper (Docker container used in PiCTURE) [27]	pysam version 0.19.1 (linux/amd64: SHA256 hash: 027eab43717917325172e1d71384883d4e17552823d28afdea92ffec4df68beb, linux/arm64: SHA256 hash: 85c7774051b295124cb8e3dcbd332bf02b80a7efd9b988b279482b9ec09892a1)	Accessed on 2025-12-10	BSD 3-Clause "New" or "Revised" License	Free via Docker Hub website (https://hub.docker.com/r/kazukinakamae/pysam)
Software	motif_extraction (Docker container used in PiCTURE)	Version 1.0 (linux/amd64: SHA256 hash: f530f9e5d0cb2a2918b2dc071eb2c49db296dac1be67bf02447170c3619d514a, linux/arm64: SHA256 hash: 6f06b3938db074f1797719cc436b50774ec27f308039ec5b412687006c108ad5)	Accessed on 2025-12-10	MIT License	Free via Docker Hub website (https://hub.docker.com/r/kazukinakamae/motif_extraction)
Software	STAR index container (Docker container used in PiCTURE)	STAR version 2.7.4a [28], Docker image version 1.0 (SHA256 hash: ba4617f902a7d900c912ad84208944cb36dd57ae3cca125ecb534756e537e12b)	Accessed on 2025-12-10	MIT License	Built locally by preparation.sh in PiCTURE
Software	MEME suites (Conda package used in promoter_dataset_generator) [29]	Version 5.5.9 (SHA256 hash: cb4fa670c3cbd498241e6e9e476754b898f8705a03e023f2aca0d122198a64d5)	Accessed on 2025-12-10	Custom license (https://github.com/cinquin/MEME?tab=License-1-ov-file#readme)	Free for non-profit Purposes (https://conda.anaconda.org/bioconda/linux-64/meme-5.5.9-pl5321h1ca524f_0.conda)
Software	fastq-dump (SRA Toolkit [15])	Version 3.0.1 (Git commit hash: 674e7b317eec5e47f9677d7e5b8f2e4ca2808514)	Accessed on 2025-12-10	Public Domain	Free via GitHub website (https://github.com/ncbi/sra-tools/releases/tag/3.0.1)
Dataset	GATK resource bundle for hg38 (GRCh38.p14 + dbSNP138 [30])	Homo_sapiens_assembly38.fasta (SHA256 hash: 93157a161863464c9435062fd67c173fdaf99cb8b32f1455018361387ffa5564) resources-broad-hg38-v0-Homo_sapiens_assembly38.dict (SHA256 hash: 07523b4a0afd1127ae909f92f8b3911aff096461aed7eff3f1acf5bdfc1dc103) resources-broad-hg38-v0-Homo_sapiens_assembly38.fasta.fai (SHA256 hash: edefd93c489dc1baefad312f40388089f8db5cf6dcc3ba0955669ead274e8b6b) resources-broad-hg38-v0-Homo_sapiens_assembly38.dbsnp138.vcf (SHA256 hash: 0ff368a3a4e16fc539be19ddbc58996d7e9a959a50e8597a7de07e217cce8527) resources-broad-hg38-v0-Homo_sapiens_assembly38.dbsnp138.vcf.idx (SHA256 hash: d0c185e0b5b43f3c2587e01c93f0c0dc81754649c0e0de69af64defa74cb33f8) resources_broad_hg38_v0_wgs_calling_regions.hg38.interval_list (SHA256 hash: 6b240928c5b367a0fcfceb4fe04a0ca8847026e6cdfbea44ae9342bed24bc7cc)	Accessed on 2025-12-10	No license (https://gatk.broadinstitute.org/hc/en-us/community/posts/16865825134747-License-use-terms-for-GATK-resource-bundle)	Free via Broad Institute website (https://gatk.broadinstitute.org/hc/en-us/articles/360035890811-Resource-bundle), or our figshare (https://doi.org/10.6084/m9.figshare.31742074)
Dataset	NCBI Sequence Read Archive (SRA) FASTQ files [11]	SRR11561273_1.fastq (MD5sum hash: d8b44bd60b4854febdc6893c7b259c5d) SRR11561273_2.fastq (MD5sum hash: f7685993088b8908e67e8f5d01063f81) SRR11561289_1.fastq (MD5sum hash: 2e8039102e8a6fdace188ec00945aae3) SRR11561289_2.fastq (MD5sum hash: 045b98ecec8d4a3b1f258634f5011384) SRR11561292_1.fastq (MD5sum hash: 4e64b30c2ea84790ade1afe9c54c4bba) SRR11561292_2.fastq (MD5sum hash: f8f6e21b2b296b085187852facab484c) SRR11561298_1.fastq (MD5sum hash: ddfa3a569bc3a34faad4dfc5400d0e1b) SRR11561298_2.fastq (MD5sum hash: 42d4dd9aba8dfb4fbd1d421b68371624) SRR11561314_1.fastq (MD5sum hash: c64cc259347666c474d5b7ae14a24437) SRR11561314_2.fastq (MD5sum hash: abfe5940d0fbf79178d81b9dd4cab8db) SRR11561324_1.fastq (MD5sum hash: 19a2455a4e94a91f3b3fb4aa360f922d) SRR11561324_2.fastq (MD5sum hash: ac1c31c0dbdd8d7c914d11ec4c94ea3a) SRR11561326_1.fastq (MD5sum hash: e3acaaf6eb448b623bbab58566bc8aef) SRR11561326_2.fastq (MD5sum hash: 7db849ec149f4944de0f37ae3fec4aca)		Custom license (https://www.ncbi.nlm.nih.gov/home/about/policies/)	Free via NCBI website (https://www.ncbi.nlm.nih.gov/sra)
Dataset	EPDnew promoter dataset for eukaryotes [16]	*Arabidopsis thaliana*: Version 004 *Zea mays*: Version 001 *Hordeum vulgare*: Version 000 *Homo sapiens*: Version 000 *Macaca mulatta*: Version 000 *Mus musculus*: Version 000 *Rattus norvegicus*: Version 000 *Gallus gallus*: Version 000 *Canis familiaris*: Version 000 *Drosophila melanogaster*: Version 000 *Apis mellifera*: Version 000 *Danio rerio*: Version 000 *Caenorhabditis elegans*: Version 000 *Saccharomyces cerevisiae*: Version 000 *Schizosaccharomyces pombe*: Version 000	Accessed on 2025-12-03	Creative Commons Attribution 4.0 International Public License (CC BY 4.0)	Free via EPD website (https://epd.expasy.org/epd/EPDnew_database.php)

To improve computational reproducibility, we recorded the operating system and GPU driver versions used for the protocol. PiCTURE was executed on Ubuntu 22.04.5 LTS (Linux kernel: 5.15.0-118-generic, NVIDIA driver: version 550.54.14, CUDA runtime: version 12.4, No CUDA Toolkit [31], Docker: version 27.5.1). DNABERT-2 fine-tuning was executed on Ubuntu 22.04.5 LTS with an NVIDIA H100 (80 GB) (Linux kernel: Linux 5.15.0-163-generic, NVIDIA driver: version 580.95.05, CUDA runtime: version 13.0, CUDA Toolkit: version 12.2). The other data processing tasks were executed using only CPU resources on macOS 26.0 (Darwin kernel: version 25.2.0).

DNABERT-2 was fine-tuned using Transformers 4.29.2 and PyTorch 2.4.1 (CUDA 12.1 build). The NVIDIA driver reported CUDA 13.0 support via nvidia-smi, and CUDA Toolkit 12.2 was installed for development and optional compilation tasks.

**RNA-seq dataset**

RNA-seq data were used in the PiCTURE pipeline. RNA-seq datasets were downloaded from the NCBI Sequence Read Archive (SRA). In this protocol, data for BE4-rAPOBEC1–transfected samples (SRR11561324, SRR11561298, and SRR11561273), BE4-RrA3F–transfected sample (SRR11561292), and nCas9-transfected samples (SRR11561289, SRR11561314, and SRR11561326) [11] were downloaded in FASTQ format using the fastq-dump utility from the SRA Toolkit.

## Procedure

**A. Constructing labeled DNA sequence datasets**

**A1. Preparation for the RNA off-target dataset using the *PiCTURE* pipeline**

**1. Installation of *PiCTURE***

The PiCTURE pipeline can be installed via GitHub (https://github.com/KazukiNakamae/PiCTURE). The *preparation.sh* in the repository downloads the dataset and Docker images required for its execution.

*Note: The memory should be at least 100 GB for efficient execution.*

Use the following code to install PiCTURE (comments are provided below #) after opening a new terminal session on the Linux Server:

# Get PiCTURE scripts

git clone https://github.com/KazukiNakamae/PiCTURE.git;

# Prepare the PiCTURE Pipeline and dataset using preparation.sh

cd PiCTURE/PiCTURE/Docker;

chmod +x preparation.sh

bash preparation.sh $(date +%Y%m%d%H%M%S)_working_directory

**Critical:** If you are not in the docker group, use sudo.

**Critical:** Approximately 10 GB of storage is required, and the analysis typically takes about half a day to complete.

# Example

sudo bash preparation.sh $(date +%Y%m%d%H%M%S)_working_directory;

PiCTURE's preparation.sh automatically downloads the human reference genome resources from Broad Institute's hg38 resource bundle hosted in Google Cloud Public Datasets (base URL: https://storage.googleapis.com/genomics-public-data/resources/broad/hg38/v0/). The downloaded files include Homo_sapiens_assembly38.fasta (and .dict/.fai), dbsnp_138.hg38.vcf (and index), and the wgs_calling_regions interval list. No authentication is required for these HTTPS downloads. The script also downloads docker image of *clinicalgenomics/trim_galore:0.6.7, broadinstitute/gatk:4.3.0.0, kazukinakamae/motif_extraction:1.0, staphb/bcftools:1.16, mgibio/picard-cwl:2.18.1*, and *ewels/multiqc:v1.14, kazukinakamae/pysam:0.19.1*, and builds the Docker image for STAR mapping using the hg38 index. The *bam_preparation_v2.sh* is generated.

We suggest integrity check commands right after running *preparation.sh*.

head -n 1 Homo_sapiens_assembly38.fasta

# Here is the expected response.

>chr1 AC:CM000663.2 gi:568336023 LN:248956422 rl:Chromosome M5:6aef897c3d6ff0c78aff06ac189178dd AS:GRCh38

sha256sum Homo_sapiens_assembly38.fasta;

# Here is the expected response.

93157a161863464c9435062fd67c173fdaf99cb8b32f1455018361387ffa5564

sha256sum 20251209121256_working_directory/4_bam_preparation/resources-broad-hg38-v0-Homo_sapiens_assembly38.dict

# Here is the expected response.

07523b4a0afd1127ae909f92f8b3911aff096461aed7eff3f1acf5bdfc1dc103

sha256sum 20251209121256_working_directory/4_bam_preparation/resources-broad-hg38-v0-Homo_sapiens_assembly38.fasta.fai

# Here is the expected response.

edefd93c489dc1baefad312f40388089f8db5cf6dcc3ba0955669ead274e8b6b

sha256sum 20251209121256_working_directory/5_recal_data/resources-broad-hg38-v0-Homo_sapiens_assembly38.dbsnp138.vcf

# Here is the expected response.

0ff368a3a4e16fc539be19ddbc58996d7e9a959a50e8597a7de07e217cce8527

sha256sum 20251209121256_working_directory/5_recal_data/resources-broad-hg38-v0-Homo_sapiens_assembly38.dbsnp138.vcf.idx

# Here is the expected response.

d0c185e0b5b43f3c2587e01c93f0c0dc81754649c0e0de69af64defa74cb33f8

sha256sum 20251209121256_working_directory/7a_single_genomics_dbImport/resources_broad_hg38_v0_wgs_calling_regions.hg38.interval_list

# Here is the expected response.

6b240928c5b367a0fcfceb4fe04a0ca8847026e6cdfbea44ae9342bed24bc7cc

sha256sum 20251209121256_working_directory/4_bam_preparation/bam_preparation_v2.sh

# Here is the expected response.

134a37cf319e545be72b5599c62219c8948a3ac48baeae279444a32ec2d4e946

We also provide a copy of resource bundle files in figshare (https://doi.org/10.6084/m9.figshare.31742074).

**2. Genome mapping of RNA-seq data from CBE-treated and control (e.g., nCas9) samples to the human genome reference (hg38) using the PiCTURE pipeline**

PiCTURE receives paired-end RNA-seq data (.fastq) as input. RNA-seq datasets from CBE-treated (SRR11561324, SRR11561298, SRR11561273, and SRR11561292) and control (nCas9-transfected) samples (SRR11561289, SRR11561314, and SRR11561326) can be obtained from the NCBI Sequence Read Archive (SRA). Place the data in the current directory (*PiCTURE/PiCTURE/Docker*).

We have placed the data below in the current directory for this demonstration (Table 2).

**Table 2.** Map between sample type and RNA-seq data (R1/R2)

Sample type	R1	R2
CBE (BE4-rAPOBEC1)	SRR11561324_1.fastq	SRR11561324_2.fastq
CBE (BE4-rAPOBEC1)	SRR11561298_1.fastq	SRR11561298_2.fastq
CBE (BE4-rAPOBEC1)	SRR11561273_1.fastq	SRR11561273_2.fastq
CBE (BE4-RrA3F)	SRR11561292_1.fastq	SRR11561292_2.fastq
Control (nCas9)	SRR11561289_1.fastq	SRR11561289_2.fastq
Control (nCas9)	SRR11561314_1.fastq	SRR11561314_2.fastq
Control (nCas9)	SRR11561326_1.fastq	SRR11561326_2.fastq

**Critical:** PiCTURE expects uncompressed paired-end FASTQ files (.fastq). Gzipped FASTQ (.fastq.gz) is not supported; if your data are compressed, decompress them before running PiCTURE. The large disk space (>100 GB RAM, significant storage) is necessary for the *bash run.sh script*. If you are not in the docker group, use sudo.

The FASTQ files are downloaded using the following commands:

fastq-dump --split-files SRR11561324

fastq-dump --split-files SRR11561298

fastq-dump --split-files SRR11561273

fastq-dump --split-files SRR11561292

fastq-dump --split-files SRR11561289

fastq-dump --split-files SRR11561314

fastq-dump --split-files SRR11561326

We suggest running integrity checks immediately after downloading the FASTQ files.

md5sum *.fastq

# Here was the expected response.

d8b44bd60b4854febdc6893c7b259c5d SRR11561273_1.fastq

f7685993088b8908e67e8f5d01063f81 SRR11561273_2.fastq

2e8039102e8a6fdace188ec00945aae3 SRR11561289_1.fastq

045b98ecec8d4a3b1f258634f5011384 SRR11561289_2.fastq

4e64b30c2ea84790ade1afe9c54c4bba SRR11561292_1.fastq

f8f6e21b2b296b085187852facab484c SRR11561292_2.fastq

ddfa3a569bc3a34faad4dfc5400d0e1b SRR11561298_1.fastq

42d4dd9aba8dfb4fbd1d421b68371624 SRR11561298_2.fastq

c64cc259347666c474d5b7ae14a24437 SRR11561314_1.fastq

abfe5940d0fbf79178d81b9dd4cab8db SRR11561314_2.fastq

19a2455a4e94a91f3b3fb4aa360f922d SRR11561324_1.fastq

ac1c31c0dbdd8d7c914d11ec4c94ea3a SRR11561324_2.fastq

e3acaaf6eb448b623bbab58566bc8aef SRR11561326_1.fastq

7db849ec149f4944de0f37ae3fec4aca SRR11561326_2.fastq

Here is the FASTQ file example:

@SRR11561324.1 1 length=150

CNGGTGTAAAGGTATCTGCTGCATCGAACTTTAAACTTCACGTTGTCCTTATTTTTCTTGATCTTGACAGATTTGGCATCCTTTCGTCGGGCTGTGAGCAGGAAGTCCTTGATTTCCTCAATTTTCCGAGGCATGGCGACGAGGCGCGCT

+SRR11561324.1 1 length=150

F#FFFFFFFFFFFFFFFFFFFFFFFFFFFFFFFFFFFFFFFFFFFFF:FFFFFFFFFFFFF:FFFFFFFFFFFFFFFFF,FFFFFFFFFFFFFFFFFFFFFFFFFFFFFFFFFFFFFFFFFFFFFFFFFFFFFFF:FFFFFFFF:F:FFF

@SRR11561324.2 2 length=150

GNGGAAAGACGCAGCAATTTGCCAGGAGGTCAAGCCCACCAATTTCGGGGATCTGCTGTGCACACCGGGTTCCTTCTTAATCCCTGCTGAGGATCTTGAGATCGGAAGAGCACACGTCTGAACTCCAGTCACATTCAGAAATCGCGTATG

+SRR11561324.2 2 length=150

F#FF:FFFFFFFFFFFFFFFFFFFFFFFFFFFFFFFFFFFFFFFFFFFFFFFFFFFFFFFFFFFFFFFFFFFFFFFFFFFFFFFFFFFFFFFFFFFFFFFFFFFFFFFFFFFFFFFFFFFFFFFFFFFFFFFFFFFFFFFFFF,FFFFFF

Here is the current directory structure:

Docker

├── Dockerfile

├── Homo_sapiens_assembly38.fasta

├── SRR11561273_1.fastq

├── SRR11561273_2.fastq

├── SRR11561289_1.fastq

├── SRR11561289_2.fastq

├── SRR11561292_1.fastq

├── SRR11561292_2.fastq

├── SRR11561298_1.fastq

├── SRR11561298_2.fastq

├── SRR11561314_1.fastq

├── SRR11561314_2.fastq

├── SRR11561324_1.fastq

├── SRR11561324_2.fastq

├── SRR11561326_1.fastq

├── SRR11561326_2.fastq

├── get_intersection_variants.sh

├── get_result.sh

├── get_result_from_singledb.sh

├── motif_estimation.sh

├── preparation.sh

├── run.sh

├── variant_identification_from_multidb.sh

└── variant_identification_from_singledb.sh

Use the following commands as examples to run *PiCTURE* (comments are provided following #) on the Linux Server:

# Example

# bash run.sh [R1.fastq] [R2.fastq] [Sample name] [Working directory] [Maximum memory];

# CBE (BE4-rAPOBEC1)

bash run.sh \

SRR11561324_1.fastq \

SRR11561324_2.fastq \

SRR11561324 \

20251209121256_working_directory \

120;

bash run.sh \

SRR11561298_1.fastq \

SRR11561298_2.fastq \

SRR11561298 \

20251209121256_working_directory \

120;

bash run.sh \

SRR11561273_1.fastq \

SRR11561273_2.fastq \

SRR11561273 \

20251209121256_working_directory \

120;

# CBE (BE4-RrA3F)

bash run.sh \

SRR11561292_1.fastq \

SRR11561292_2.fastq \

SRR11561292 \

20251209121256_working_directory \

120;

# Control (nCas9)

bash run.sh \

SRR11561289_1.fastq \

SRR11561289_2.fastq \

SRR11561289 \

20251209121256_working_directory \

120;

bash run.sh \

SRR11561314_1.fastq \

SRR11561314_2.fastq \

SRR11561314 \

20251209121256_working_directory \

120;

bash run.sh \

SRR11561326_1.fastq \

SRR11561326_2.fastq \

SRR11561326 \

20251209121256_working_directory \

120;

**Critical:** If you are not in the docker group, use sudo.

**Critical:** Approximately 250 GB of storage is required, and the analysis typically takes about seven days to complete.

Here is the working directory structure:

20251209121256_working_directory

├── 0_rawdata

├── 1_trim_galore

├── 2_star

├── 3_twopass_align

├── 4_bam_preparation

├── 5_recal_data

├── 6_haplotypecaller

├── 7a_single_genomics_dbImport

├── 7b_joint_genomics_dbImport

├── joint_db

└── single_db

The directories in the working directory contain intermediate files, not the results. Briefly describe the files in each directory:

*0_rawdata*: The directory is generated to temporarily store input fastq files. The fastq files have been deleted when finishing run.sh script, so it is empty.

*1_trim_galore*: The results of trimming and QC of fastq files and their reports were saved to the directory. The fastq files were deleted upon completion of the run.sh script, so it contains only QC reports.

*2_star*: The directory contained the results of STAR alignment, including the alignment BAM file (*Aligned.sortedByCoord.out.bam*), splice junction information (*SJ.out.tab*), a summary of the alignment results (*Log.final.out*), and the log file (*Log.out*).

*3_twopass_align*: The directory contains the output of the two-pass alignment performed with STAR [32]. The files are largely the same as those in the *2_star* directory, including *Aligned.sortedByCoord.out.bam*, with additional auxiliary files and directories such as *Log.progress.out* and *STARgenome* also generated.

*4_bam_preparation*: The directory stores the results of post-processing applied to the output BAM file. These steps include adding read group information, marking duplicates, and splitting reads that contain Ns in their CIGAR strings. The processed BAM file is saved as *[Sample name].complete.bam*.

*5_recal_data*: The directory contains the results of base quality score recalibration (BQSR) [33] performed using dbSNP138 for hg38 as the set of known SNPs. In addition to the recalibrated BAM file (*dbsnp_only.BQSR.bam*), the recalibration tables (*dbsnp_only_post_recal_data.table* and *dbsnp_only_recal_data.table*) and recalibration reports (*recalibration_dbsnp_only_plots.csv* and *recalibration_dbsnp_only_plots.pdf*) are also generated.

**3. Variant calling and sequence motif extraction**

The *PiCTURE* performs variant calling and generates a variant call format (VCF: format version v4.2) file from a single RNA-seq dataset using *GATK haplotypecaller* [22,23,34]. The pipeline comprises three substitution strategies: (i) single-sample genotyping, (ii) joint genotyping, and (iii) intersection.

The VCF files are saved to the *8_vcf_identification* directory. Here is the VCF file example:

##fileformat=VCFv4.2

(The header lines are omitted because it is too long)

#CHROM POS ID REF ALT QUAL FILTER INFO FORMAT SRR11561324

chr1 14464 . A T 260.04 . AC=2;AF=1.00;AN=2;DP=8;ExcessHet=0.0000;FS=0.000;MLEAC=2;MLEAF=1.00;MQ=60.00;QD=32.50;SOR=2.833 GT:AD:DP:GQ:PL 1/1:0,8:8:24:274,24,0

chr1 14542 . A G 159.64 . AC=1;AF=0.500;AN=2;BaseQRankSum=3.71;DP=22;ExcessHet=0.0000;FS=2.671;MLEAC=1;MLEAF=0.500;MQ=60.00;MQRankSum=0.00;QD=7.26;ReadPosRankSum=1.07;SOR=2.110 GT:AD:DP:GQ:PL 0/1:16,6:22:99:167,0,367

chr1 14574 . A G 397.64 . AC=1;AF=0.500;AN=2;BaseQRankSum=0.242;DP=43;ExcessHet=0.0000;FS=15.086;MLEAC=1;MLEAF=0.500;MQ=60.00;MQRankSum=0.00;QD=9.25;ReadPosRankSum=1.01;SOR=3.967 GT:AD:DP:GQ:PL 0/1:26,17:43:99:405,0,685

**(i) Single-sample genotyping**

The *variant_identification_from_singledb.sh* of PiCTURE generates a VCF file from a single RNA-seq dataset. The following commands should be used on the Linux Server:

# Example

# bash variant_identification_from_singledb.sh [Sample name] [Working directory];

# CBE (BE4-rAPOBEC1)

bash variant_identification_from_singledb.sh \

SRR11561324 \

20251209121256_working_directory;

bash variant_identification_from_singledb.sh \

SRR11561298 \

20251209121256_working_directory;

bash variant_identification_from_singledb.sh \

SRR11561273 \

20251209121256_working_directory;

# CBE (BE4-RrA3F)

bash variant_identification_from_singledb.sh \

SRR11561292 \

20251209121256_working_directory;

# Control (nCas9)

bash variant_identification_from_singledb.sh \

SRR11561289 \

20251209121256_working_directory;

bash variant_identification_from_singledb.sh \

SRR11561314 \

20251209121256_working_directory;

bash variant_identification_from_singledb.sh \

SRR11561326 \

20251209121256_working_directory;

**(ii) Joint genotyping**

The *variant_identification_from_multidb.sh* of PiCTURE generates a VCF file from multiple RNA-Seq datasets. The following commands should be used on the Linux Server:

# Example

# bash variant_identification_from_multidb.sh [Working directory] [Group name] [Sample name] … [Sample name]

# CBE (BE4-rAPOBEC1)

bash variant_identification_from_multidb.sh \

20251209121256_working_directory \

JG_BE4_rAPOBEC1 \

SRR11561324 \

SRR11561298 \

SRR11561273;

# Control (nCas9)

bash variant_identification_from_multidb.sh \

20251209121256_working_directory \

JG_nCas9 \

SRR11561289 \

SRR11561314 \

SRR11561326;

**(iii) Intersection**

The *get_intersection_variants.sh* function of PiCTURE extracts shared variants across multiple VCF files after generating outputs from strategies (i) and (ii) for at least two datasets. The following commands should be used on the Linux Server:

# Example

# bash get_intersection_variants.sh [Working directory] [Set name] [Sample name or group name] [Sample name or group name]

# Extract the common mutations between two single genotyping results of BE4-rAPOBEC1

bash get_intersection_variants.sh \

20251209121256_working_directory \

SET_SRR11561324_AND_SRR11561298 \

SRR11561324 \

SRR11561298;

**Critical:** If you are not in the docker group, use sudo.

The results are saved to *6_haplotypecaller, 7a_single_genomics_dbImport, single_db, 7b_joint_genomics_dbImport*, and *joint_db* directories.

The *6_haplotypecaller* directory contains variant calls generated by *GATK HaplotypeCaller* and stored in GVCF format. From the output, single-sample genotyping is performed using the *resources_broad_hg38_v0_wgs_calling_regions.hg38.interval_list* stored in *7a_single_genomics_dbImport* and the GenomicsDB (*single_db*) constructed from the corresponding GVCF files. The resulting VCF files are stored in *8_vcf_identification* as *[Sample name].hg38.identified.vcf*.

In addition, joint genotyping is performed using the *[Group name].sample.map* and *resources_broad_hg38_v0_wgs_calling_regions.hg38.interval_list* stored in *7b_joint_genomics_dbImport* and the GenomicsDB (*joint_db*) constructed from the corresponding GVCF files. The resulting VCF files are stored as *[Group name].hg38.identified.vcf*.

Furthermore, multiple selected *[Sample|Group name].hg38.identified.vcf* files from *8_vcf_identification* are merged and stored as *[Set name].merge.vcf* in the same directory. Records corresponding only to duplicated variants are extracted and saved as *[Set name].hg38.identified.vcf* in *8_vcf_identification*.

**4. Substitution detection and sequence motif extraction in high-frequency mutations**

*PiCTURE* can extract high-frequency substitutions and generate this sequence motif using the *motif_estimation.sh* function of *PiCTURE*. Mutations can also be selected based on variant allele frequency (VAF).

**Critical:** Substitution sites located at high frequencies are not necessarily suitable as training data. To determine whether to use the data, it is preferable to confirm the distribution of mutation data in control samples extracted using the same VAF threshold. In this protocol, we did not use sequence sets filtered by VAF, and all output substitution sequences were used in the downstream analysis. If a clear difference in the VAF distribution is observed between the control and treated samples, it is recommended to define an appropriate threshold based on the difference and use the partitioned data as the training dataset. Such preprocessing can make the results of downstream motif analysis clearer, while also enabling more efficient removal of noisy data and potentially facilitating model training.

Use the following commands in the Linux Server:

# Example

# bash motif_estimation.sh [Sample|Group|Set name] [Working directory] [VAF threshold]

# Single-sample genotyping;CBE (BE4-rAPOBEC1)

bash motif_estimation.sh \

SRR11561324 \

20251209121256_working_directory \

0.8;

bash motif_estimation.sh \

SRR11561298 \

20251209121256_working_directory \

0.8;

bash motif_estimation.sh \

SRR11561273 \

20251209121256_working_directory \

0.8;

# Single-sample genotyping;CBE (BE4-RrA3F) for comparison (not used in fine-tuning)

bash motif_estimation.sh \

SRR11561292 \

20251209121256_working_directory \

0.8;

# Single-sample genotyping; Control (nCas9)

bash motif_estimation.sh \

SRR11561289 \

20251209121256_working_directory \

0.8;

bash motif_estimation.sh \

SRR11561314 \

20251209121256_working_directory \

0.8;

bash motif_estimation.sh \

SRR11561326 \

20251209121256_working_directory \

0.8;

# Joint genotyping; CBE (BE4-rAPOBEC1)

bash motif_estimation.sh \

JG_BE4_rAPOBEC1 \

20251209121256_working_directory \

0.8;

# Joint genotyping; Control (nCas9)

bash motif_estimation.sh \

JG_nCas9 \

20251209121256_working_directory \

0.8;

# Intersection; Two datasets of single-sample genotyping;CBE (BE4-rAPOBEC1)

bash motif_estimation.sh \

SET_SRR11561324_AND_SRR11561298 \

20251209121256_working_directory \

0.8;

**Critical:** If you are not in the docker group, use sudo.

The results of all substitution sites were saved to *9_snp_detection, 10_snp_hard_filter, 11_snp_classification*, and *12_motif_extraction* directories.

The VCF files containing only the substitution records were in the *9_snp_detection*.

The hard-filtering VCF files were in the *10_snp_hard_filter*. Here is the filtering criteria:

*QD < 2.0*

*QUAL < 30.0*

*SOR > 3.0*

*FS > 60.0*

*MQ < 40.0*

*MQRankSum < -12.5*

*ReadPosRankSum < -8.0*

The genomic sequences of mutation sites classified by substitution type are saved to the *11_snp_classification*. Each VCF file is saved to the *[Sample|Group|Set name].hg38.identified.snp.fltr.[Original base]to[Substituted base].fa.*

The motif analysis results (*[Sample|Group|Set name].hg38.identified.snp.fltr.[Original base]to[Substituted base].fa.png* and *([Sample|Group|Set name].hg38.identified.snp.fltr.[Original base]to[Substituted base].fa.txt*) are in the *12_motif_extraction*.

*[Sample|Group|Set name].hg38.identified.snp.fltr.[Original base]to[Substituted base].fa.png* shows the sequence logo [35] of ±50 bp flanking regions around the detected variant sites.

*[Sample|Group|Set name].hg38.identified.snp.fltr.[Original base]to[Substituted base].fa.txt* shows base probabilities and information content in bits. Here is an example:

## LogoData

# First column is position number, counting from zero

# Subsequent columns are raw symbol counts

# Entropy is mean entropy measured in nats.

# Low and High are the 95% confidence limits.

# Weight is the fraction of non-gap symbols in the column.

#

# A C G T Entropy Low High Weight

1 12176 11300 10851 12568 0.0017 0.0012 0.0023 1.0000

2 12207 11216 10736 12736 0.0023 0.0017 0.0029 1.0000

(…)

51 0 46895 0 0 1.3859 1.3854 1.3865 1.0000

(…)

100 12480 11170 10851 12394 0.0019 0.0014 0.0025 1.0000

101 12475 11155 10969 12296 0.0017 0.0011 0.0022 1.0000

# End LogoData

The motif analysis results of substitution records split by VAF threshold are saved to *[Sample|Group|Set name].hg38.identified.snp.fltr.vaf.headerfixed.0.0_[VAF threshold]vaf* and *[Sample|Group|Set name].hg38.identified.snp.fltr.vaf.headerfixed.[VAF threshold]_1.0vaf* directories. The contents were the same as the *12_motif_extraction* directory above.

**5. Export data**

*PiCTURE* can export report files (.tar.gz) into *[Working directory]/report* directory using *get_result.sh* from *PiCTURE*. The following commands should be used on the Linux Server:

# Example

# bash get_result.sh [Sample|Group|Set name] [Working directory] [Output name];

# Single-sample genotyping;CBE (BE4-rAPOBEC1)

bash get_result.sh \

SRR11561324 \

20251209121256_working_directory \

SG_BE4_rAPOBEC1_SRR11561324;

bash get_result.sh \

SRR11561298 \

20251209121256_working_directory \

SG_BE4_rAPOBEC1_SRR11561298;

bash get_result.sh \

SRR11561273 \

20251209121256_working_directory \

SG_BE4_rAPOBEC1_SRR11561273;

# Single-sample genotyping;CBE (BE4-RrA3F)

bash get_result.sh \

SRR11561292 \

20251209121256_working_directory \

SG_BE4_RrA3F_SRR11561292;

# Single-sample genotyping; Control (nCas9)

bash get_result.sh \

SRR11561289 \

20251209121256_working_directory \

SG_BE4_nCas9_SRR11561289;

bash get_result.sh \

SRR11561314 \

20251209121256_working_directory \

SG_BE4_nCas9_SRR11561314;

bash get_result.sh \

SRR11561326 \

20251209121256_working_directory \

SG_BE4_nCas9_SRR11561326;

# Joint genotyping; CBE (BE4-rAPOBEC1)

bash get_result.sh \

JG_BE4_rAPOBEC1 \

20251209121256_working_directory \

JG_BE4_rAPOBEC1;

# Joint genotyping; Control (nCas9)

bash get_result.sh \

JG_nCas9 \

20251209121256_working_directory \

JG_nCas9;

# Intersection; Two datasets of single-sample genotyping;CBE (BE4-rAPOBEC1)

bash get_result.sh \

SET_SRR11561324_AND_SRR11561298 \

20251209121256_working_directory \

SET_SRR11561324_AND_SRR11561298;

**Critical:** If you are not in the docker group, use sudo.

Here is the report directory structure:

*[Working directory]*/report/[Output name]

├── all

│   ├── figure

│   ├── motif

│   ├── sequence

│   ├── summary.txt

│   └── vcf

└── each

 ├── JG_BE4_rAPOBEC1.hg38.identified.snp.fltr.vaf.headerfixed.0.0_0.8vaf

 │   ├── figure

 │   ├── motif

 │   ├── sequence

 │   └── vcf

 ├── JG_BE4_rAPOBEC1.hg38.identified.snp.fltr.vaf.headerfixed.0.8_1.0vaf

 │   ├── figure

 │   ├── motif

 │   ├── sequence

 │   └── vcf

 └── summary.txt

The results of all substitution sites are saved to all directories. The results of the substitution records, split by VAF threshold, are saved to *each* directory.

The VCF files are saved to the *vcf* directory. The genomic sequences of the mutation sites are saved to the *sequence* directory. The motif analysis result is saved to the *motif* directory. The *figure* directory contains the *MultiQC* report of the VCF file. The *summary.txt* is also the vcf report exported from *bcftools stats*.

**6. Data transfer of PiCTURE outputs**

The subsequent steps can be processed on a client PC, such as a MacBook Pro, after data transfer of the report directory using *rsync*. The output files from PiCTURE are saved in the report directory with the name “[Sample/Group/Set Name]. tar.gz,” which can be extracted.

rsync --partial --size-only -avhP user@XXX.XX.XXX.XX:~/XXX/[working_directory]/report XXX/;

for a in $(ls report); do tar -zxvf report/${a} -C report;done;

**7. Setting positive and negative datasets**

We define positive datasets as substitution sites detected in CBE samples and negative datasets as sites detected in nCas9 samples for fine-tuning. The fine-tuned dataset pairs are listed in Table 3.

**Table 3.** Fine-tuned dataset pairs for detecting CBE-specific RNA off-targets

Data name	Positive dataset	Negative dataset
FD1	SG_BE4_rAPOBEC1_SRR11561324, SG_BE4_rAPOBEC1_SRR11561298, SG_BE4_rAPOBEC1_SRR11561273	SG_BE4_nCas9_SRR11561289, SG_BE4_nCas9_SRR11561314, SG_BE4_nCas9_SRR11561326
FD2	JG_BE4_rAPOBEC1	JG_nCas9
FD3	SET_SRR11561324_AND_SRR11561298	JG_nCas9
FD4	SG_BE4_RrA3F_SRR11561292	JG_nCas9

For each sample, the fixed-sequences (101 bp) whose window is centered on cytosine where a C-to-U (T) substitution is detected are saved in *[Working directory]/report/[Dataset name]/all/sequence/[Dataset name].hg38.identified.snp.fltr.CtoT.fa*. The sequence is cut to the desired training sequence length (e.g., 40 bp) without shifting its center position. The following commands are used:

# Build the environment for data processing

conda env create -f ../../scripts/fine-tuning/MethodB/ft_processing_env.yml;

conda activate ft_processing_env;

# Prepare directories

for i in {1..4}

do

mkdir -p FD${i}/positive;

mkdir -p FD${i}/negative;

done;

# Copy sequences to FD1 directories

seqkit subseq -r 31:70 report/SG_BE4_rAPOBEC1_SRR11561324/all/sequence/SRR11561324.hg38.identified.snp.fltr.CtoT.fa > FD1/positive/40bp_SG_BE4_rAPOBEC1_SRR11561324.hg38.identified.snp.fltr.CtoT.fa;

seqkit subseq -r 31:70 report/SG_BE4_rAPOBEC1_SRR11561298/all/sequence/SRR11561298.hg38.identified.snp.fltr.CtoT.fa > FD1/positive/40bp_SG_BE4_rAPOBEC1_SRR11561298.hg38.identified.snp.fltr.CtoT.fa;

seqkit subseq -r 31:70 report/SG_BE4_rAPOBEC1_SRR11561273/all/sequence/SRR11561273.hg38.identified.snp.fltr.CtoT.fa > FD1/positive/40bp_SG_BE4_rAPOBEC1_SRR11561273.hg38.identified.snp.fltr.CtoT.fa;

seqkit subseq -r 31:70 report/SG_BE4_nCas9_SRR11561289/all/sequence/SRR11561289.hg38.identified.snp.fltr.CtoT.fa > FD1/negative/40bp_SG_BE4_nCas9_SRR11561289.hg38.identified.snp.fltr.CtoT.fa;

seqkit subseq -r 31:70 report/SG_BE4_nCas9_SRR11561314/all/sequence/SRR11561314.hg38.identified.snp.fltr.CtoT.fa > FD1/negative/40bp_SG_BE4_nCas9_SRR11561314.hg38.identified.snp.fltr.CtoT.fa;

seqkit subseq -r 31:70 report/SG_BE4_nCas9_SRR11561326/all/sequence/SRR11561326.hg38.identified.snp.fltr.CtoT.fa > FD1/negative/40bp_SG_BE4_nCas9_SRR11561326.hg38.identified.snp.fltr.CtoT.fa;

# Copy sequences to FD2 directories

seqkit subseq -r 31:70 report/JG_BE4_rAPOBEC1/all/sequence/JG_BE4_rAPOBEC1.hg38.identified.snp.fltr.CtoT.fa > FD2/positive/40bp_JG_BE4_rAPOBEC1.hg38.identified.snp.fltr.CtoT.fa;

seqkit subseq -r 31:70 report/JG_nCas9/all/sequence/JG_nCas9.hg38.identified.snp.fltr.CtoT.fa > FD2/negative/40bp_JG_nCas9.hg38.identified.snp.fltr.CtoT.fa;

# Copy sequences to FD3 directories

seqkit subseq -r 31:70 report/SET_SRR11561324_AND_SRR11561298/all/sequence/SET_SRR11561324_AND_SRR11561298.hg38.identified.snp.fltr.CtoT.fa > FD3/positive/40bp_SET_SRR11561324_AND_SRR11561298.hg38.identified.snp.fltr.CtoT.fa;

seqkit subseq -r 31:70 report/JG_nCas9/all/sequence/JG_nCas9.hg38.identified.snp.fltr.CtoT.fa > FD3/negative/40bp_JG_nCas9.hg38.identified.snp.fltr.CtoT.fa;

# Copy sequences to FD4 directories

seqkit subseq -r 31:70 report/SG_BE4_RrA3F_SRR11561292/all/sequence/SRR11561292.hg38.identified.snp.fltr.CtoT.fa > FD4/positive/40bp_SG_BE4_RrA3F_SRR11561292.hg38.identified.snp.fltr.CtoT.fa;

seqkit subseq -r 31:70 report/JG_nCas9/all/sequence/JG_nCas9.hg38.identified.snp.fltr.CtoT.fa > FD4/negative/40bp_JG_nCas9.hg38.identified.snp.fltr.CtoT.fa;

*Note: The length of the sequences used for training should ideally reflect the biological mechanism of the prediction target. The SpCas9 protein used in the BE4 system typically recognizes a ~20 bp protospacer together with a 3 bp NGG PAM [8], resulting in a total target region of approximately 23 bp. Because the deaminase activity window peaks within the protospacer region, we estimated that, even when considering a broader range, the region that could physically interact with the editing complex would extend at most to about twice the length. Therefore, we set the training sequence length to 40 bp. For genome-editing tools that act over a broader range, this sequence window may need to be reevaluated.*

**8. Merge dataset**

To fine-tune the dataset pair, the positive and negative datasets are merged into a single sequence file (.fa). The following commands should be used:

for i in {1..4}

do

cat FD${i}/positive/*.fa > FD${i}/positive_40bp.fa;

cat FD${i}/negative/*.fa > FD${i}/negative_40bp.fa;

done;

**9. Removing duplicate sequences within the dataset**

To prevent the same sequence from appearing in the dataset, sequences that overlapped within the positive or negative datasets should be removed.

*Note: Because sequences may coincidentally become identical even if extracted from different genomic loci, this process must be performed after cutting the sequences to the desired training length (Section A1.7).*

Use the following commands:

for i in {1..4}

do

seqkit rmdup -s < FD${i}/positive_40bp.fa > FD${i}/positive_40bp.rmdup.fa;

seqkit rmdup -s < FD${i}/negative_40bp.fa > FD${i}/negative_40bp.rmdup.fa;

done;

**10. Filtering and input dataset creation**

*PiCTURE/scripts*/fine-tuning/*MethodB/create_ft_data_dnabert2_20240704_all.py* constructs the training, validation, and test datasets by excluding sequences from the positive dataset that are common to both the positive and negative datasets. The input dataset of DNABERT-2 consists of three CSV files: train.csv, dev.csv, and test.csv, each with a header sequence, label, and one sequence per row, where the label is 1 for positive and 0 for negative.

*Note: The results of this step can also be included in the training dataset for fine-tuning DNABERT-2; however, balancing in the next step is preferred.*

Use the following commands:

# Example

# python ../../scripts/fine-tuning/MethodB/create_ft_data_dnabert2_20240704_all.py --positive [Positive data] --negative [Negative data] --seed [Random seed] --outdir [Output directory];

for i in {1..4}

do

mkdir FD${i}/dataset_v1_union_40bp;

python ../../scripts/fine-tuning/MethodB/create_ft_data_dnabert2_20240704_all.py \

--positive FD${i}/positive_40bp.rmdup.fa \

--negative FD${i}/negative_40bp.rmdup.fa \

--seed 0 \

--outdir FD${i}/dataset_v1_union_40bp;

done;

*Note: The input FASTA sequences should be provided in uppercase and restricted to the ATCG nucleotides. Ambiguous bases are not expected to be included.*

Here is an example of the CSV file:

*sequence,label*

*CATAGTTATCAGTGGGGCCACACTGCCCTTTAGATATGTA,1*

*ATTAAGATCAAGGATACTGACTGAAATATGAATTTGCTCA,1*

*CAGGCAGCAGTCTCCAGAGACAACACTGGAGCCATCGTAG,1*

*CCATAAGCATTTACCATTTACTTAATATTTGCCATCAGCA,1*

*GTCTAGGCAGCATAGTGAGACTCCATCTCTACAATTTTTT,1*

The *sequence* column is an uppercase DNA string (A/C/G/T). The *label* column is a binary integer (0/1).

**11. Data balancing using undersampling**

The *balance_labels.py* performs undersampling on the training, validation, and test datasets to address the imbalance between the positive and negative datasets. Because class imbalance in the labeled samples can lead to training instability and bias in evaluation metrics, the dataset should be balanced as much as possible through undersampling.　The output directory comprises the final dataset used in the DNABERT-2 fine-tuning. The following commands should be used:

*Notes:*

*1. In the scripts we provide, a seed value can be specified using the --seed option. To reproduce the same sampling results, this seed value must be set to the same value.*

*2. Changing the seed option generates replicate datasets that can be used for fine-tuning. However, because the underlying sequence data overlap across replicates, it is preferable to increase the RNA-seq dataset size and re-run the pipeline from Section A1.2 for fairer dataset construction when additional RNA-seq data suitable for biological replicates are available.*

# Example

# python balance_labels.py --train [Training dataset] --dev [Validation dataset] --test [Test dataset] --seed [Random seed] --output_dir [Output directory];

for i in {1..4}

do

mkdir FD${i}/dataset_v1_union_40bp_balanced;

python ../../scripts/fine-tuning/MethodB/balance_labels.py \

--train FD${i}/dataset_v1_union_40bp/train.csv \

--dev FD${i}/dataset_v1_union_40bp/dev.csv \

--test FD${i}/dataset_v1_union_40bp/test.csv \

--seed 1 \

--output_dir FD${i}/dataset_v1_union_40bp_balanced;

done;

**12. Check the number of labels of the fine-tuned dataset for CBE-specific RNA off-target prediction**

Use the following commands. The count result is described in Table 4.

for d in FD*; do

 [ -d "$d/dataset_v1_union_40bp_balanced" ] || continue

 echo "== $d =="

 for f in "$d"/dataset_v1_union_40bp_balanced/{train,dev,test}.csv; do

 [ -f "$f" ] || continue

 awk -F, -v file="$(basename "$f")" '

 FNR==1{

 for(i=1;i<=NF;i++){ gsub(/\r/,"",$i); if($i=="label") lc=i }

 next

 }

 {

 v=$(lc)

 gsub(/\r/,"",v)

 gsub(/^[ \t]+|[ \t]+$/,"",v)

 if(v=="0"||v=="1") c[v]++

 }

 END{ printf("%s\t0: %d\t1: %d\n", file, c["0"]+0, c["1"]+0) }

 ' "$f"

 done

 echo

done

**Table 4.** Sample number of the fine-tuning dataset pairs for detecting CBE-specific RNA off-targets

Data name	Dataset	Positive labels	Negative labels
FD1	Train	31,880	31,880
	Development	6,831	6,831
	Test	6,832	6,832
FD2	Train	32,368	32,368
	Development	6,936	6,936
	Test	6,937	6,937
FD3	Train	3,656	3,656
	Development	7,84	784
	Test	7,84	784
FD4	Train	7,229	7,229
	Development	1,549	1,549
	Test	1,550	1,550

**A2. Construct promoter datasets**

Promoter information should be collected for each class of organisms, as shown in Table 5. In this section, the datasets are processed using the same steps.

**Table 5.** Target taxonomic groups and the species included in the promoter dataset

Target taxonomic group of promoter sequences	Species
Mammal	*Homo sapiens, Macaca mulatta, Mus musculus, Rattus norvegicus, Canis familiaris*
Mammal (ncRNA)	*Homo sapiens, Mus musculus*
Bird	*Gallus gallus*
Insect	*Drosophila melanogaster, Apis mellifera*
Fish	*Danio rerio*
Nematoda	*Caenorhabditis elegans*
Plant	*Arabidopsis thaliana, Zea mays, Hordeum vulgare*
Yeast	*Saccharomyces cerevisiae, Schizosaccharomyces pombe*

**1. Download positive promoter sequences from *EPDnew***

Eukaryotic promoter sequences should be obtained from *EPDnew* and stored locally as positive datasets for each species.

Use the following commands to clone the repository and prepare the working directory:

git clone https://github.com/KazukiNakamae/mammal_promoter_classifier.git;

cd mammal_promoter_classifier;

# Make directory to store EPDnew promoter FASTA files

mkdir EPDnewDB;

Download promoter sequences from *EPDnew* as follows:

a. Access the EPDnew database page: https://epd.expasy.org/epd/EPDnew_database.php.

b. Open the selection/download tool: https://epd.expasy.org/epd/EPDnew_select.php.

c. For each species of interest, set the extraction window relative to the transcription start site (TSS) and export the promoter sequences in the FASTA format.

d. Save all downloaded FASTA files into the *EPDnewDB* directory created above.

*Note: Using this protocol, we obtained promoter regions from −499 bp to +200 bp around the annotated TSSs.*

**2. Build the Docker environment for promoter dataset generation**

A Docker image containing all the required tools should be built from the Dockerfile in the repository.

Use the following command to build the image:

docker build --platform=linux/amd64 \

-t kazukinakamae/promoter_dataset_generator:1.0 \

-f Dockerfile .

**Critical:** If you are not in the docker group, use sudo.

The image contains the MEME suite (v5.5.9) [29]. MEME Suite is distributed under an academic/non-commercial license (free for educational, research, and non-profit use; commercial use may require a license; see the MEME Suite copyright/licensing information).

**3. Generate negative (non-promoter) datasets by di-nucleotide shuffling**

Negative datasets should be generated by shuffling each positive promoter sequence while preserving the dinucleotide composition using fasta-shuffle letters from the MEME suite (v5.5.9). The following commands are used:

*Note: Randomized sequences used as a negative dataset may lose not only promoter-specific features but also broader characteristics of genomic sequences. If negative examples that retain genomic properties are desired, it is preferable to use alternative datasets, such as experimentally validated inactive promoter sequences, rather than randomized sequences.*

mkdir negatives;

for f in EPDnewDB/*.fa;

do

bn=$(basename "$f" .fa);

docker run --platform=linux/amd64 --rm \

-v `pwd`:/DATA -w /DATA \

-i kazukinakamae/promoter_dataset_generator:1.0 \

fasta-shuffle-letters -k 2 -seed 1 "$f" "negatives/${bn}_neg.fa";

done;

As an alternative, di-nucleotide shuffling can be performed using *shuffle_fasta_di.py* if MEME Suite is unavailable.

# Alternative method

# Download shuffle_fasta_di.py

git clone https://github.com/KazukiNakamae/shuffle_fasta_di.git;

# di-nucleotide shuffling

mkdir negatives;

for f in EPDnewDB/*.fa;

do

bn=$(basename "$f" .fa);

python shuffle_fasta_di/shuffle_fasta_di.py "$f" "negatives/${bn}_neg.fa" 1;

done;

**4. Construct training, validation, and test datasets**

Positive and negative promoter sequences across species should be merged, labeled, cleaned, and split into training, validation, and test sets using the *make_datasets.py script*.

First, create a CSV file that maps the *EPDnew* file identifiers to species labels. The first column must match the base names of the FASTA files in the *EPDnewDB* (without the .fa extension), and the second column provides species labels to be used for stratified splitting.

cat << EOF > species_map.csv

hg38_xg1A3,Human

mm10_Urwkp,Mouse

rheMac8_v0ZSy,Rhesus_monkey

rn6_fZYbR,Rat

canFam3_VCC6C,Dog

EOF

Subsequently, *make_datasets.py* reads positive FASTA files from *EPDnewDB* and negative FASTA files from the negatives. It then drops sequences containing ambiguous bases (N) and converts them to uppercase. Finally, the script assigns species labels based on species_map.csv and splits the data into training, validation, and test sets based on species labels.

docker run --platform=linux/amd64 --rm \

-v `pwd`:/DATA -w /DATA \

-i kazukinakamae/promoter_dataset_generator:1.0 \

python make_datasets.py \

--pos EPDnewDB \

--neg negatives \

--out_dir dataset \

--expect_len 700 --drop_N --uppercase \

--test_size 0.10 --dev_size 0.10 --seed 1 \

--stratify species_label \

--species_map species_map.csv;

The outputs should comprise three CSV files from the dataset directory: train.csv for training, dev.csv for validation, and test.csv for testing.

Each CSV comprises two columns: sequence (700 bp promoter or shuffled sequence) and label (1 for positive promoter sequences, 0 for shuffled negative sequences), which are directly usable as inputs for DNABERT-2 fine-tuning.

**5. Check the number of labels for the fine-tuned dataset for promoter prediction**

Use the following commands. The count result is described in Table 6.

for d in *_promoter_classifier; do

 [ -d "$d/dataset" ] || continue

 echo "== $d =="

 for f in "$d"/dataset/{train,dev,test}.csv; do

 [ -f "$f" ] || continue

 awk -F, -v file="$(basename "$f")" '

 FNR==1{

 for(i=1;i<=NF;i++){ gsub(/\r/,"",$i); if($i=="label") lc=i }

 next

 }

 {

 v=$(lc)

 gsub(/\r/,"",v)

 gsub(/^[ \t]+|[ \t]+$/,"",v)

 if(v=="0"||v=="1") c[v]++

 }

 END{ printf("%s\t0: %d\t1: %d\n", file, c["0"]+0, c["1"]+0) }

 ' "$f"

 done

 echo

done

**Table 6.** Sample number of the fine-tuned dataset pairs for predicting the promoter

Data name	Dataset	Positive labels	Negative labels
Mammal	Train	66,568	66,570
	Development	8,322	8,321
	Test	8,322	8,321
Mammal (ncRNA)	Train	4,331	4,331
	Development	542	542
	Test	542	542
Bird	Train	4,900	4,900
	Development	612	612
	Test	612	612
Insect	Train	18,582	18,582
	Development	2,322	2,322
	Test	2,322	2,322
Fish	Train	8,547	8,547
	Development	1,068	1,068
	Test	1,068	1,068
Nematoda	Train	5,696	5,696
	Development	712	712
	Test	712	712
Plant	Train	48,613	48,615
	Development	6,077	6,076
	Test	6,077	6,076
Yeast	Train	7,935	7,935
	Development	992	992
	Test	992	992

**B. Fine-tuning DNABERT-2 on custom datasets**

Fine-tuning using DNABERT-2 requires a high-performance GPU. In this section, we perform the following tasks using Sakura's Cloud Server with NVIDIA H100:

**B1. Set up the software environment of the GPU cloud**

**1. Prepare and mount the NVMe SSD for data storage**

The attached NVMe SSD should be configured as a high-throughput data volume for the temporary data. The device should be formatted using the XFS file system and mounted under */mnt/nvme*. Furthermore, its availability should be confirmed from the operating system. The following commands were used:

# Update package lists and install NVMe utilities

sudo apt-get update

sudo apt-get install -y nvme-cli

# List NVMe devices and identify the target (e.g., /dev/nvme0n1)

nvme list

# Format the NVMe device with XFS

sudo mkfs -t xfs /dev/nvme0n1

# Create a mount point and mount the NVMe volume

sudo mkdir -p /mnt/nvme

sudo mount /dev/nvme0n1 /mnt/nvme

# Confirm that the NVMe volume is mounted with the XFS filesystem

df -Th

**2. Verify that the NVIDIA GPU is detected by the system**

Check whether the GPU is correctly exposed as a PCI device before installing and configuring the driver and CUDA toolkit [31]. This ensures that the hardware is visible at the OS level. The following commands were used:

# Confirm that the NVIDIA GPU is detected on the PCI bus

lspci | grep -i nvidia

**3. Install and configure Git for downloading DNABERT-2 and helper scripts**

Install Git from the Ubuntu repositories and configure the global username and email address. This enables the cloning of DNABERT-2 and custom helper repositories. The following commands should be used:

# Install Git

sudo apt-get install -y git

# Confirm installation

dpkg -l git

# Set global Git user information

git config --global user.name "XXX"

git config --global user.email "XXX@XXX"

**4. Install Miniforge and prepare the Conda environment manager**

Miniforge (a minimal Conda distribution from Conda-forge) should be installed to manage isolated Python environments for DNABERT-2 fine-tuning and dependency control. The following commands should be used:

# Download and install Miniforge (Linux x86_64)

wget https://github.com/conda-forge/miniforge/releases/latest/download/Miniforge3-Linux-x86_64.sh

bash Miniforge3-$(uname)-$(uname -m).sh

Open a new shell window and initialize the Miniforge environment as instructed by the installer.

**5. Synchronize kernel headers and build environment for the NVIDIA driver**

Ensure that the running Linux kernel, headers, and build tools are consistent. This is required for proper installation. The following commands should be used:

*# Check the current kernel version*

*uname -r*

*# Reinstall the kernel image, headers, extra modules, and build toolchain*

*sudo apt-get update*

*sudo apt-get install --reinstall \*

*linux-image-$(uname -r) \*

*linux-headers-$(uname -r) \*

*linux-modules-extra-$(uname -r) \*

*build-essential dkms*

**6. Install the Ubuntu-packaged NVIDIA driver and verify GPU status**

The Ubuntu-provided NVIDIA driver (via Ubuntu drivers) should be used to avoid conflicts between the OS and vendor drivers. After installation and rebooting, confirm that the GPU is recognized and that the Nvidia-SMI reports the device correctly. The following commands should be used:

# Install the recommended NVIDIA driver from Ubuntu repositories

sudo apt-get update

sudo ubuntu-drivers autoinstall

# (Alternatively: sudo apt-get install nvidia-driver-535)

# Reboot to load the NVIDIA driver

sudo reboot

# After reboot, verify that the GPU and driver are functional

nvidia-smi

**Pause point:** If *nvidia-smi* does not work after driver installation and reboot, the installed driver package, kernel module status, and kernel header consistency should be rechecked.

**7. Install CUDA Toolkit 12.2 without modifying the NVIDIA driver**

Install the NVIDIA CUDA Toolkit 12.2 from the official CUDA APT repository while retaining the Ubuntu-managed driver. PATH and LD_LIBRARY_PATH are updated so that CUDA 12.2 tools (including nvcc) are available without altering the driver version. The following commands should be used:

# Register the NVIDIA CUDA APT repository (Ubuntu 22.04 / x86_64 example)

cd /tmp

wget https://developer.download.nvidia.com/compute/cuda/repos/ubuntu2204/x86_64/cuda-keyring_1.0-1_all.deb

sudo dpkg -i cuda-keyring_1.0-1_all.deb

sudo apt-get update

# Install CUDA Toolkit 12.2 only (no driver change)

sudo apt-get install -y cuda-toolkit-12-2

# Add CUDA Toolkit 12.2 to PATH and LD_LIBRARY_PATH

echo 'export PATH=/usr/local/cuda-12.2/bin${PATH:+:${PATH}}' >> ~/.bashrc

echo 'export LD_LIBRARY_PATH=/usr/local/cuda-12.2/lib64${LD_LIBRARY_PATH:+:${LD_LIBRARY_PATH}}' >> ~/.bashrc

source ~/.bashrc

# Confirm the CUDA compiler version

nvcc -V

---

nvcc: NVIDIA (R) Cuda compiler driver

Copyright (c) 2005-2023 NVIDIA Corporation

Built on Tue_Aug_15_22:02:13_PDT_2023

Cuda compilation tools, release 12.2, V12.2.140

Build cuda_12.2.r12.2/compiler.33191640_0

---

**Pause point:** If *nvcc* is not found after installation, review the PATH and LD_LIBRARY_PATH settings and reload the shell. It should also be noted that the CUDA version shown by nvidia-smi reflects the maximum CUDA capability supported by the installed driver, whereas *nvcc -V* reports the version of the locally installed CUDA toolkit; therefore, a discrepancy between the two outputs does not necessarily indicate an error.

**8. Clone DNABERT-2 and helper repositories**

The DNABERT-2 implementation built by Northwestern University MAGICS Labs and our unofficial helper scripts from GitHub in the home directory should be used for model fine-tuning and environmental health checks. The following commands should be used:

cd ~

git clone https://github.com/MAGICS-LAB/DNABERT_2.git

cd DNABERT_2

git clone -b v1.0.0 https://github.com/KazukiNakamae/DNABERT_2_helper.git

**9. Create and activate the Conda environment for DNABERT-2**

A dedicated conda environment (*dna*) should be created using Python 3.8, which matches the DNABERT-2 codebase and dependency requirements. The following commands should be used:

# Create and activate a Conda environment for DNABERT-2

conda create -n dna conda-forge::python=3.8

conda activate dna

**10. Run OS-level GPU/CUDA health check**

Before installing PyTorch, verify that the GPU, NVIDIA driver, and CUDA libraries are correctly configured at the OS level using a custom health-check shell script *DNABERT_2_helper/gpu_cuda_health_check.sh*. The setting is correct if the output message “No critical issues detected at OS level for GPU/CUDA” is shown.

# Run an OS-level GPU / CUDA health check

bash DNABERT_2_helper/gpu_cuda_health_check.sh

**11. Install PyTorch (CUDA 12.1 build) and verify GPU support**

Upgrade the PIP and install official PyTorch, TorchVision, and TorchAudio binaries built for CUDA 12.1. Although CUDA Toolkit 12.2 is installed system-wide, it includes its own compatible CUDA runtime (cu121). A custom Python script, *DNABERT_2_helper/torch_health_check.py*, should be used to confirm that PyTorch can detect and use the H100 GPU. The setting is correct if the output message ”No critical issues are detected” is shown.

# Install PyTorch (CUDA 12.1 build) and related packages

pip3 install --upgrade pip

pip3 install torch torchvision torchaudio --index-url https://download.pytorch.org/whl/cu121

# Verify PyTorch / CUDA integration

python DNABERT_2_helper/torch_health_check.py

**12. Install remaining Python dependencies for DNABERT-2**

After confirming that PyTorch [19] and Triton [36] function correctly, the remaining Python packages (e.g., Transformers [20], PEFT [37], and other utilities) should be obtained from a modified requirements file tailored for DNABERT-2 fine-tuning.

# Install remaining dependencies, including Transformers and utilities

pip3 install -r DNABERT_2_helper/modified_requirements.txt

**13. Adjust Triton installation for DNABERT-2 compatibility**

The original DNABERT-2 implementation depended on Triton version *2.0.0.dev20221202*, which is incompatible with Triton 3.x. When the environment is dedicated to DNABERT-2, the Triton 3.x should be removed. In this configuration, DNABERT-2 can run without Triton at the cost of slightly reduced speed and memory efficiency but with a negligible impact on model accuracy. The following commands are used:

# Remove Triton 3.x to avoid version conflicts with DNABERT-2

pip uninstall -y triton

**B2. Configure DNABERT-2 fine-tuning**

**1. Data transfer of datasets**

Transfer the train.csv, dev.csv, and test.csv files for a given task into a single directory from the client PC (MacBook Pro) to Sakura's cloud server using an H100 GPU.

rsync -e "ssh -i ~/.ssh/XXX -p XXX" --partial --size-only -avhP [dataset directory] user@XX.XXXX.XX.XXX:~/;

**2. Check the number of available GPUs**

Check the number of available GPUs and enter the value for the num_gpu environmental variable. The following commands should be used:

nvidia-smi -L;

# In this case,

# GPU 0: NVIDIA H100 80GB …

export num_gpu=1

**3. Check the configuration of the parameters of *train.py***

The *train.py* can perform the fine-tuning of DNABERT-2. We recommend using the officially distributed *zhihan1996/DNABERT-2-117M* pretrained model as the basis for fine-tuning. The maximum sequence length *(--model_max_length*) was set based on the sequence length (e.g., 10 for 40-bp sequences), following the recommendation that the tokenized length should be ~4× smaller than the nucleotide length.

**4. Grid search for optimizing hyperparameters**

Adjust various hyperparameters of train.py, primarily the training batch size (--per_device_train_batch_size), gradient accumulation (--gradient_accumulation_steps), learning rate (--learning_rate), epochs (--num_train_epochs), weight decay (--weight_decay), and warmup (--warmup_steps), which will lead to optimal learning results. These parameters are selected because they have a direct and often interacting influence on training stability, convergence speed, generalization performance, and GPU memory usage. In DNABERT-2, as in other transformer-based models, model performance can be highly sensitive to the balance between optimization efficiency and regularization, so systematically exploring these hyperparameters is a practical way to identify a robust training configuration.

The per-device training batch size (--per_device_train_batch_size) is included because it determines how many samples are processed at once on each device, which affects both memory consumption and the stability of gradient estimates. Larger batch sizes can provide smoother optimization and faster throughput, but they require more GPU memory and may sometimes reduce generalization. Smaller batch sizes are more memory-efficient and can introduce beneficial gradient noise, although they may also make training less stable.

Gradient accumulation steps (--gradient_accumulation_steps) are examined alongside batch size, as they control the effective batch size without requiring additional GPU memory. By accumulating gradients over multiple forward and backward passes before updating the model, it becomes possible to simulate a larger batch size even under hardware constraints. The parameter, therefore, affects optimization stability, update frequency, and the trade-off between computational efficiency and memory limitations.

The learning rate (--learning_rate) is one of the most important parameters because it strongly influences how quickly and how reliably the model converges. A learning rate that is too high can cause unstable training or divergence, whereas a learning rate that is too low can slow convergence and lead to suboptimal solutions. For transformer-based models such as DNABERT-2, careful tuning of the learning rate is especially important, as fine-tuning performance is often highly sensitive to it.

The number of training epochs (--num_train_epochs) is also selected because it controls how long the model is exposed to the training data. Too few epochs may result in underfitting, where the model has not learned enough from the data, while too many epochs may lead to overfitting, where performance on the training set improves but generalization to unseen data deteriorates. Searching over the number of epochs helps identify an appropriate training duration for the specific dataset and task.

Weight decay (--weight_decay) is included as a regularization parameter to reduce overfitting and improve generalization. It works by penalizing overly large parameter values during optimization, which can help the model learn smoother and more robust representations. In practice, the optimal amount of weight decay depends on the size of the dataset, the complexity of the task, and the extent to which the model is prone to memorizing training examples.

Warmup steps (--warmup_steps) are selected because they are particularly important in transformer training. During the warmup phase, the learning rate is gradually increased from a small initial value to the target learning rate, which helps avoid unstable updates at the beginning of training. This is especially useful when fine-tuning pretrained models such as DNABERT-2, where abrupt large updates early in training can damage useful pretrained representations.

Our helper scripts provide both subset creation and grid search programs for hyperparameter optimization. The *DNABERT_2_helper/subset_dataset_csv.py* creates a subset for grid search.

*Note: When the available dataset is small, we recommend increasing the subset sampling proportion specified by the subset_ratio option.*

BASE_DIR=${HOME}

# Subset creation for fine-tuning for the RNA off-target task using FD1

python DNABERT_2_helper/subset_dataset_csv.py \

--input_dir ${BASE_DIR}/FD1/dataset_v1_union_40bp_balanced \

--output_dir ${BASE_DIR}/FD1/dataset_v1_union_40bp_balanced_subset20 \

--subset_ratio 0.2 \

--seed 1 \

--label_column label;

python DNABERT_2_helper/subset_dataset_csv.py \

--input_dir ${BASE_DIR}/FD2/dataset_v1_union_40bp_balanced \

--output_dir ${BASE_DIR}/FD2/dataset_v1_union_40bp_balanced_subset20 \

--subset_ratio 0.2 \

--seed 1 \

--label_column label;

python DNABERT_2_helper/subset_dataset_csv.py \

--input_dir ${BASE_DIR}/FD3/dataset_v1_union_40bp_balanced \

--output_dir ${BASE_DIR}/FD3/dataset_v1_union_40bp_balanced_subset20 \

--subset_ratio 0.2 \

--seed 1 \

--label_column label;

python DNABERT_2_helper/subset_dataset_csv.py \

--input_dir ${BASE_DIR}/FD4/dataset_v1_union_40bp_balanced \

--output_dir ${BASE_DIR}/FD4/dataset_v1_union_40bp_balanced_subset20 \

--subset_ratio 0.2 \

--seed 1 \

--label_column label;

# Subset creation for fine-tuning for the promoter prediction using mammal dataset

python DNABERT_2_helper/subset_dataset_csv.py \

--input_dir ${BASE_DIR}/mammal_promoter_classifier/dataset \

--output_dir ${BASE_DIR}/mammal_promoter_classifier/dataset_subset20 \

--subset_ratio 0.2 \

--seed 1 \

--label_column label;

The *DNABERT_2_helper/grid_search_train_wrapper.sh* performs grid search.

*Note: For a grid search, it is advisable to explore a parameter range as wide as feasible. In particular, the use of a broad set of learning rate values increases the likelihood of identifying high-performance hyperparameter combinations.*

cd finetune;

# Grid search for fine-tuning for the RNA off-target prediction using FD1

bash ../DNABERT_2_helper/grid_search_train_wrapper.sh \

--train_script train.py \

--data_path ${BASE_DIR}/FD1/dataset_v1_union_40bp_balanced_subset20 \

--model_name_or_path zhihan1996/DNABERT-2-117M \

--output_root ${BASE_DIR}/DNABERT_2/finetune/fd1_rnaofftarget_grid_subset20 \

--run_name_prefix fd1_rnaofftarget \

--nproc_per_node ${num_gpu} \

--batch_configs "8x8,16x8,32x8" \

--lrs "1e-5,2e-5" \

--epochs "2" \

--warmup_ratios "0.05,0.1" \

--weight_decays "0.01,0.03" \

--extra_args "--kmer -1 --model_max_length 10 --fp16 --find_unused_parameters False"

# Get the best hyperparameters for fine-tuning for RNA off-target prediction using FD1

python ../DNABERT_2_helper/select_best_run.py \

--output_root ${BASE_DIR}/DNABERT_2/finetune/fd1_rnaofftarget_grid_subset20 \

--metric_name eval_loss \

--metric_mode min \

--num_train_epochs 8 \

--train_script ${BASE_DIR}/DNABERT_2/finetune/train.py \

--model_name_or_path zhihan1996/DNABERT-2-117M \

--data_path ${BASE_DIR}/FD1/dataset_v1_union_40bp_balanced \

--print_full_command;

# Grid search for fine-tuning for the RNA off-target prediction using FD2

bash ../DNABERT_2_helper/grid_search_train_wrapper.sh \

--train_script train.py \

--data_path ${BASE_DIR}/FD2/dataset_v1_union_40bp_balanced_subset20 \

--model_name_or_path zhihan1996/DNABERT-2-117M \

--output_root ${BASE_DIR}/DNABERT_2/finetune/fd2_rnaofftarget_grid_subset20 \

--run_name_prefix fd2_rnaofftarget \

--nproc_per_node ${num_gpu} \

--batch_configs "8x8,16x8,32x8" \

--lrs "1e-5,2e-5" \

--epochs "2" \

--warmup_ratios "0.05,0.1" \

--weight_decays "0.01,0.03" \

--extra_args "--kmer -1 --model_max_length 10 --fp16 --find_unused_parameters False"

The *DNABERT_2_helper/select_best_run.py* selects the best combination of hyperparameters in terms of eval loss.

*Note: If a particular performance metric (accuracy, F1 score, Matthews correlation coefficient [38], precision, or recall) is of primary interest, it can be specified via the “--metric_name” option, such that the best hyperparameter combination is shown based on that metric rather than the eval loss.*

# Get the best hyperparameters for fine-tuning for RNA off-target prediction using FD2

python ../DNABERT_2_helper/select_best_run.py \

--output_root ${BASE_DIR}/DNABERT_2/finetune/fd2_rnaofftarget_grid_subset20 \

--metric_name eval_loss \

--metric_mode min \

--num_train_epochs 8 \

--train_script ${BASE_DIR}/DNABERT_2/finetune/train.py \

--model_name_or_path zhihan1996/DNABERT-2-117M \

--data_path ${BASE_DIR}/FD2/dataset_v1_union_40bp_balanced \

--print_full_command;

# Grid search for fine-tuning for the RNA off-target prediction using FD3

bash ../DNABERT_2_helper/grid_search_train_wrapper.sh \

--train_script train.py \

--data_path ${BASE_DIR}/FD3/dataset_v1_union_40bp_balanced_subset20 \

--model_name_or_path zhihan1996/DNABERT-2-117M \

--output_root ${BASE_DIR}/DNABERT_2/finetune/fd3_rnaofftarget_grid_subset20 \

--run_name_prefix fd3_rnaofftarget \

--nproc_per_node ${num_gpu} \

--batch_configs "8x8,16x8,32x8" \

--lrs "1e-5,2e-5" \

--epochs "2" \

--warmup_ratios "0.05,0.1" \

--weight_decays "0.01,0.03" \

--extra_args "--kmer -1 --model_max_length 10 --fp16 --find_unused_parameters False"

# Get the best hyperparameters for fine-tuning for RNA off-target prediction using FD3

python ../DNABERT_2_helper/select_best_run.py \

--output_root ${BASE_DIR}/DNABERT_2/finetune/fd3_rnaofftarget_grid_subset20 \

--metric_name eval_loss \

--metric_mode min \

--num_train_epochs 8 \

--train_script ${BASE_DIR}/DNABERT_2/finetune/train.py \

--model_name_or_path zhihan1996/DNABERT-2-117M \

--data_path ${BASE_DIR}/FD3/dataset_v1_union_40bp_balanced \

--print_full_command;

# Grid search for fine-tuning for the RNA off-target prediction using FD4

bash ../DNABERT_2_helper/grid_search_train_wrapper.sh \

--train_script train.py \

--data_path ${BASE_DIR}/FD4/dataset_v1_union_40bp_balanced_subset20 \

--model_name_or_path zhihan1996/DNABERT-2-117M \

--output_root ${BASE_DIR}/DNABERT_2/finetune/fd4_rnaofftarget_grid_subset20 \

--run_name_prefix fd4_rnaofftarget \

--nproc_per_node ${num_gpu} \

--batch_configs "8x8,16x8,32x8" \

--lrs "1e-5,2e-5" \

--epochs "2" \

--warmup_ratios "0.05,0.1" \

--weight_decays "0.01,0.03" \

--extra_args "--kmer -1 --model_max_length 10 --fp16 --find_unused_parameters False"

# Get the best hyperparameters for fine-tuning for RNA off-target prediction using FD4

python ../DNABERT_2_helper/select_best_run.py \

--output_root ${BASE_DIR}/DNABERT_2/finetune/fd4_rnaofftarget_grid_subset20 \

--metric_name eval_loss \

--metric_mode min \

--num_train_epochs 8 \

--train_script ${BASE_DIR}/DNABERT_2/finetune/train.py \

--model_name_or_path zhihan1996/DNABERT-2-117M \

--data_path ${BASE_DIR}/FD4/dataset_v1_union_40bp_balanced \

--print_full_command;

The same step can also be applied to fine-tune the promoter classification.

# Grid search for fine-tuning for the promoter prediction using mammal dataset

bash ../DNABERT_2_helper/grid_search_train_wrapper.sh \

--train_script train.py \

--data_path ${BASE_DIR}/mammal_promoter_classifier/dataset_subset20 \

--model_name_or_path zhihan1996/DNABERT-2-117M \

--output_root ${BASE_DIR}/DNABERT_2/finetune/mammal_promoter_grid_subset20 \

--run_name_prefix mammal_promoter \

--nproc_per_node ${num_gpu} \

--batch_configs "8x8,16x8,32x8" \

--lrs "1e-5,2e-5" \

--epochs "2" \

--warmup_ratios "0.05,0.1" \

--weight_decays "0.01,0.03" \

--extra_args "--kmer -1 --model_max_length 175 --fp16 --find_unused_parameters False"

# Get the best hyperparameters for fine-tuning for the promoter prediction using mammal dataset

python ../DNABERT_2_helper/select_best_run.py \

--output_root ${BASE_DIR}/DNABERT_2/finetune/mammal_promoter_grid_subset20 \

--metric_name eval_loss \

--metric_mode min \

--num_train_epochs 8 \

--train_script ${BASE_DIR}/DNABERT_2/finetune/train.py \

--model_name_or_path zhihan1996/DNABERT-2-117M \

--data_path ${BASE_DIR}/mammal_promoter_classifier/dataset \

--print_full_command;

We then applied a grid search to the other datasets. *Select_best_run. py* suggests the best hyperparameters for each dataset (Table 7). We fixed the number of epochs at eight for the final model training.

**Table 7.** Optimal hyperparameter combinations identified by grid search for each dataset

Dataset	Train batch size	Gradient accumulation	Learning rate	Epochs	Weight decay	Warmup
FD1 RNA offtarget	16	8	2×10^-5^	8	0.01	199
FD2 RNA offtarget	8	8	2×10^-5^	8	0.03	404
FD3 RNA offtarget	8	8	2×10^-5^	8	0.03	46
FD4 RNA offtarget	8	8	1×10^-5^	8	0.01	180
Mammal Promoter	16	8	2×10^-5^	8	*0.03*	*832*
Mammal (ncRNA) Promoter	8	8	2×10^-5^	8	*0.03*	54
Bird Promoter	8	8	2×10^-5^	8	0.01	123
Insect Promoter	8	8	2×10^-5^	8	0.01	464
Fish Promoter	8	8	2×10^-5^	8	0.01	107
Nematoda Promoter	8	8	2×10^-5^	8	0.03	71
Plant Promoter	8	8	2×10^-5^	8	0.01	608
Yeast Promoter	8	8	2×10^-5^	8	0.03	198

**B3. Run fine-tuning for the RNA off-target prediction**

The FD1 CBE-specific RNA off-target dataset constructed in section A1 should be fine-tuned using the hyperparameters recommended in section B2.

# Example: FD1

mkdir fd1_output;

torchrun --nproc_per_node=${num_gpu} train.py \

--model_name_or_path zhihan1996/DNABERT-2-117M \

--data_path ${BASE_DIR}/FD1/dataset_v1_union_40bp_balanced \

--kmer -1 \

--run_name fd1_rnaofftarget \

--model_max_length 10 \

--per_device_train_batch_size 16 \

--per_device_eval_batch_size 32 \

--gradient_accumulation_steps 8 \

--learning_rate 2e-5 \

--num_train_epochs 8 \

--fp16 \

--save_steps 200 \

--output_dir ${BASE_DIR}/DNABERT_2/finetune/fd1_output \

--evaluation_strategy steps \

--eval_steps 200 \

--weight_decay 0.01 \

--warmup_steps 199 \

--overwrite_output_dir True \

--log_level info \

--find_unused_parameters False \

--save_model True;

Using the same procedure described above, CBE-specific RNA off-target prediction models should be built using the FD2-4 datasets.

**B4. Run fine-tuning for the promoter prediction**

The mammalian promoter dataset constructed in section A2 should be fine-tuned using the hyperparameters recommended in section B2.

# Example: Mammal

mkdir mpc_output;

torchrun --nproc_per_node=${num_gpu} train.py \

--model_name_or_path zhihan1996/DNABERT-2-117M \

--data_path /home/nakamae/mammal_promoter_classifier/dataset \

--kmer -1 \

--run_name dnabert2_mammal_promoter \

--model_max_length 175 \

--per_device_train_batch_size 16 \

--per_device_eval_batch_size 32 \

--gradient_accumulation_steps 8 \

--learning_rate 2e-05 \

--num_train_epochs 8 \

--fp16 \

--save_steps 200 \

--output_dir ${BASE_DIR}/DNABERT_2/finetune/mpc_output \

--evaluation_strategy steps \

--eval_steps 200 \

--weight_decay 0.03 \

--warmup_steps 832 \

--overwrite_output_dir True \

--log_level info \

--find_unused_parameters False \

--save_model True;

Using the procedure described above, promoter prediction models should be built for mammals (ncRNAs), birds, insects, fish, nematodes, plants, and yeasts.

**B5. (Optional) Resuming DNABERT-2 fine-tuning**

The *DNABERT_2_helper/resume_same_task_same_data.py* resumes fine-tuning the same task and dataset from an existing checkpoint directory. The following example shows the retraining of a classification model trained on the BE4-rAPOBEC1 RNA off-target dataset (FD1 dataset) for the same task using the FD1 dataset.

# Example: Prediction model based on FD1 dataset resumes training from checkpoint-3800 with FD1 dataset

mkdir resume_checkpoint;

cp -r ${BASE_DIR}/DNABERT_2/finetune/fd1_output/checkpoint-3800 resume_checkpoint/;

mkdir fd1_resume_from_3800_output;

torchrun --nproc_per_node=${num_gpu} ../DNABERT_2_helper/resume_same_task_same_data.py \

--checkpoint_dir ${BASE_DIR}/DNABERT_2/finetune/resume_checkpoint/checkpoint-3800 \

--data_path ${BASE_DIR}/FD1/dataset_v1_union_40bp_balanced \

--output_dir fd1_resume_from_3800_output \

--add_epochs 8 \

--do_test_eval;

**B6. (Optional) New task/new label set from an existing checkpoint**

The *train.py* can fine-tune a model for a new task, starting from a specific checkpoint of a previously fine-tuned model. The example below shows the retraining of the existing classification model, trained on the BE4-rAPOBEC1 RNA off-target dataset (FD1), to classify the RNA off-target dataset (FD4) derived from the BE4-RrA3F RNA off-targets.

# FD1 -> FD4

mkdir fd4_from_fd1_checkpoint-3800_output;

torchrun --nproc_per_node=${num_gpu} train.py \

--model_name_or_path ${BASE_DIR}/DNABERT_2/finetune/fd1_output/checkpoint-3800 \

--data_path ${BASE_DIR}/FD4/dataset_v1_union_40bp_balanced \

--kmer -1 \

--run_name fd4_from_fd1_ckpt3800 \

--model_max_length 10 \

--per_device_train_batch_size 8 \

--per_device_eval_batch_size 16 \

--gradient_accumulation_steps 8 \

--learning_rate 1e-05 \

--num_train_epochs 8 \

--fp16 \

--save_steps 200 \

--output_dir fd4_from_fd1_checkpoint-3800_output \

--evaluation_strategy steps \

--eval_steps 200 \

--weight_decay 0.01 \

--warmup_steps 180 \

--logging_steps 100 \

--overwrite_output_dir True \

--log_level info \

--find_unused_parameters False \

--save_model True;

**B7. (Optional) Baseline for promoter classification using a 3-layer one-hot convolutional neural network (CNN)**

*DNABERT_2_helper/onehot_cnn_baseline.py* performed machine learning using a 3-layer one-hot CNN [39]. This script could be used as a creation of a baseline for promoter classification.

cd ${BASE_DIR}/DNABERT_2/DNABERT_2_helper;

mkdir baseline_onehot_cnn;

python onehot_cnn_baseline.py \

 --train_csv ${BASE_DIR}/mammal_promoter_classifier/dataset/train.csv \

 --dev_csv ${BASE_DIR}/mammal_promoter_classifier/dataset/dev.csv \

 --test_csv ${BASE_DIR}/mammal_promoter_classifier/dataset/test.csv \

 --outdir baseline_onehot_cnn \

 --seq_len 700 \

 --epochs 30 \

 --batch_size 128 \

 --lr 1e-3 \

 --weight_decay 1e-4;

**B8. Data transfer of prediction models**

Transfer the directories with the prediction model files from Sakura's cloud server using the H100 GPU to a client PC (MacBook Pro). Predictions and evaluations using a trained prediction model can also be performed in CPU-only environments.

rsync -e "ssh -i ~/.ssh/XXX -p XXX" --partial --size-only -avhP user@XX.XXXX.XX.XXX:~/DNABERT_2/finetune/*_output* ./;

rsync -e "ssh -i ~/.ssh/XXX -p XXX " --partial --size-only -avhP user@XX.XXXX.XX.XXX:~/DNABERT_2/DNABERT_2_helper/baseline_onehot_cnn ./;

**C. Evaluating model performance**

**1. Set up the software environment of client PC**

In many cases, client PCs are assumed not to be equipped with high-performance GPUs. The DNABERT_2_helper repository provides a CPU-based environment and scripts for running inferences using models built using DNABERT-2. To leverage these resources, it is necessary to build a dedicated Docker image.

Execute the following command:

# Download DNABERT_2_helper repository in current directory

git clone -b v1.0.0 https://github.com/KazukiNakamae/DNABERT_2_helper.git;

# Build Docker image

docker buildx build --platform linux/amd64 \

 --build-arg ENABLE_CUDA=0 \

 -t kazukinakamae/dnabert-eval:amd64 \

 DNABERT_2_helper;

# create cache directory for Huggingface Transformers

mkdir -p .hf_cache;

**Critical:** If you are not in the docker group, use sudo.

**2. Copy hold-out datasets**

The holdout datasets used during fine-tuning (*test.csv*) should be copied into a single directory (*testdata*). For ease of identification by filename, each file should be renamed in the format *<dataset_name>_test.csv*.

# Example

mkdir testdata;

cp mammal_promoter_classifier/dataset/test.csv testdata/mammal_promoter_test.csv;

cp mammal_ncRNA_promoter_classifier/dataset/test.csv testdata/mammal_ncRNA_promoter_test.csv;

cp bird_promoter_classifier/dataset/test.csv testdata/bird_promoter_test.csv;

cp fish_promoter_classifier/dataset/test.csv testdata/fish_promoter_test.csv;

cp insect_promoter_classifier/dataset/test.csv testdata/insect_promoter_test.csv;

cp nematoda_promoter_classifier/dataset/test.csv testdata/nematoda_promoter_test.csv;

cp plant_promoter_classifier/dataset/test.csv testdata/plant_promoter_test.csv;

cp yeast_promoter_classifier/dataset/test.csv testdata/yeast_promoter_test.csv;

cp PiCTURE/PiCTURE/Docker/FD1/dataset_v1_union_40bp_balanced/test.csv testdata/fd1_rnaofftarget_test.csv;

cp PiCTURE/PiCTURE/Docker/FD2/dataset_v1_union_40bp_balanced/test.csv testdata/fd2_rnaofftarget_test.csv;

cp PiCTURE/PiCTURE/Docker/FD3/dataset_v1_union_40bp_balanced/test.csv testdata/fd3_rnaofftarget_test.csv;

cp PiCTURE/PiCTURE/Docker/FD4/dataset_v1_union_40bp_balanced/test.csv testdata/fd4_rnaofftarget_test.csv;

**3. Extract predictions on the test set**

The *DNABERT_2_helper/predict_hf_classifier_csv.py* performs inference using the model files downloaded from section B8 (DNABERT_2/finetune/*_output*). Specify the column containing the input sequences (e.g., sequence) using the text_column option. The “--max_length” option should be set to the same value as “--model_max_length” used in *train.py*.

The predicted labels and probabilities, along with the true labels, should be saved in a CSV file.

Note: Several datasets must be evaluated when multiple models are generated. We recommend running the evaluation in a loop to reduce the risk of input errors, as shown in the following example.

# Example: fd1 model

mkdir fd1_model_eval_result;

for i in testdata/*_rnaofftarget_test.csv; do

 docker run --platform=linux/amd64 --rm \

 -v `pwd`:/DATA -w /DATA \

 -v `pwd`/.hf_cache:/root/.cache/huggingface \

 -i kazukinakamae/dnabert-eval:amd64 \

 python DNABERT_2_helper/predict_hf_classifier_csv.py \

 --model_dir fd1_output \

 --input_file "testdata/$(basename "$i")" \

 --output_file fd1_model_eval_result/"$(basename "$i" .csv)"_dnabert2_test_pred.csv \

 --text_column sequence \

 --max_length 10 \

 --batch_size 32 \

 --trust_remote_code;

done;

# Example: Mammal promoter classification model

mkdir mpc_model_eval_result;

for i in "${BASE_DIR}"/testdata/*_promoter_test.csv; do

 docker run --platform=linux/amd64 --rm \

 -v `pwd`:/DATA -w /DATA \

 -v `pwd`/.hf_cache:/root/.cache/huggingface \

 -i kazukinakamae/dnabert-eval:amd64 \

 python DNABERT_2_helper/predict_hf_classifier_csv.py \

 --model_dir mpc_output \

 --input_file "testdata/$(basename "$i")" \

 --output_file mpc_model_eval_result/"$(basename "$i" .csv)"_dnabert2_test_pred.csv \

 --text_column sequence \

 --max_length 175 \

 --batch_size 32 \

 --trust_remote_code;

done;

Prediction scripts output a CSV file, to which is added “sample_id” (the unique identifier), “pred_label” (the predicted class), “prob_0” (the negative probability), and “prob_1” (positive probability) columns.

Here is an example of the csv file:

*sequence,label,sample_id,pred_label,prob_0,prob_1*

*TTTTATATTGGGTTATTTGTCATATTATTGATAACTTATG,0,0,1,0.4268121,0.5731879*

*CACAGATTCAGTAAGACGCTCAAGCCACGTCAACGGGAGG,0,1,0,0.57371837,0.42628166*

*ACTTGGTTTTTCATGCACACCGGTATAAGCTAAAGTTTAG,0,2,0,0.62741166,0.3725883*

*AGAAGAAATGGATAAATTCCCGGAGACATACACCCTCCCA,0,3,0,0.80333143,0.19666857*

*CCTGCCTCAGCCTTCCAAAGCGTTGGGATTACGGGTGTGA,0,4,0,0.7193917,0.28060827*

**4. (Optional) Generating baseline predictions with a 3-layer one-hot CNN**

*DNABERT_2_helper/predict_onehot_cnn_csv.*py performs classification using the 3-layer one-hot CNN model files downloaded from section B8 (DNABERT_2/DNABERT_2_helper/baseline_onehot_cnn/*best_model.pt*).

mkdir cnn_mpc_model_eval_result;

for i in testdata/*_promoter_test.csv; do

 docker run --platform=linux/amd64 --rm \

 -v `pwd`:/DATA -w /DATA \

 -i kazukinakamae/dnabert-eval:amd64 \

 python DNABERT_2_helper/predict_onehot_cnn_csv.py \

 --model baseline_onehot_cnn/best_model.pt \

 --input "testdata/$(basename "$i")" \

 --output cnn_mpc_model_eval_result/"$(basename "$i" .csv)"_dnabert2_test_pred.csv \

 --batch_size 256 \

 --device cpu

done;

**5. (Optional) Generating baseline predictions using motif-based rules**

*DNABERT_2_helper/predict_motif_baseline_csv.py* generates motif-based baseline outputs by specifying a simple motif sequence via “--pattern” and matching the start position via “--start.”

# Example: WCW motif

mkdir WCW_motif_eval_result;

for i in "${BASE_DIR}"/testdata/*_rnaofftarget_test.csv; do

 docker run --platform=linux/amd64 --rm \

 -v `pwd`:/DATA -w /DATA \

 -i kazukinakamae/dnabert-eval:amd64 \

 python DNABERT_2_helper/predict_motif_baseline_csv.py \

 --input "testdata/$(basename "$i")" \

 --output WCW_motif_eval_result/"$(basename "$i" .csv)"_dnabert2_test_pred.csv \

 --pattern WCW \

 --start 19

done;

**6. (Optional) Filtering test data based on motif-based predictions**

The *DNABERT_2_helper/filter_evalres_seq_label.py* enables filtering of test data based on evaluation results, such as the motif-based predictions generated in section C5. In the example below, non-WCW RNA off-target sequences that did not contain the WCW motif should be extracted and subsequently reevaluated using each model.

# Example:FD1

# Filtering test data

docker run --platform=linux/amd64 --rm \

 -v `pwd`:/DATA -w /DATA \

 -i kazukinakamae/dnabert-eval:amd64 \

 python DNABERT_2_helper/filter_evalres_seq_label.py \

 --input WCW_motif_eval_result/fd1_rnaofftarget_test_dnabert2_test_pred.csv \

 --output testdata/fd1_nonWCWrnaofftarget_test.csv \

 --pred-value 0;

# Extract predictions

for i in testdata/*_nonWCWrnaofftarget_test.csv; do

 docker run --platform=linux/amd64 --rm \

 -v `pwd`:/DATA -w /DATA \

 -v `pwd`/.hf_cache:/root/.cache/huggingface \

 -i kazukinakamae/dnabert-eval:amd64 \

 python DNABERT_2_helper/predict_hf_classifier_csv.py \

 --model_dir fd1_output \

 --input_file "testdata/$(basename "$i")" \

 --output_file fd1_model_eval_result/"$(basename "$i" .csv)"_dnabert2_test_pred.csv \

 --text_column sequence \

 --max_length 10 \

 --batch_size 32 \

 --trust_remote_code;

done;

**7. Computing performance metrics**

The *DNABERT_2_helper/evaluate_predictions_csv.py* computes the performance metrics (accuracy, F1 score, Matthews correlation coefficient, precision, and recall) for each set of prediction results. The ground-truth label set should be provided via the “--gold_file option” option, and the prediction results should be provided via the “--pred_file” option. In the present use case, because the ground-truth labels should already be included in the test dataset, the same file is specified for both options. The metrics are written to <prefix>_metrics.csv.

To facilitate downstream heatmap analysis, we recommend using a prefix such as <model_or_rule_name>_eval_<test_dataset_name>_test_dnabert2_test_pred, which enables automated parsing. For the organization, the outputs should be stored in separate directories by task group (e.g., “promoter_eval_summary,” “rnaofftarget_eval_summary,” and “non-WCWrnaofftarget_eval_summary”) that correspond to the heat maps to be generated.

# Example: Mammal promoter classification model

mkdir promoter_eval_summary;

for i in mpc_model_eval_result/*_dnabert2_test_pred.csv; do

 docker run --platform=linux/amd64 --rm \

 -v `pwd`:/DATA -w /DATA \

 -i kazukinakamae/dnabert-eval:amd64 \

 python DNABERT_2_helper/evaluate_predictions_csv.py \

 --gold_file "mpc_model_eval_result/$(basename "$i")" \

 --pred_file "mpc_model_eval_result/$(basename "$i")" \

 --out_prefix promoter_eval_summary/mpc_model_eval_"$(basename "$i" .csv)" \

 --gold_label_column label \

 --pred_label_column pred_label \

 --id_column sample_id \

 --average macro \

 --pos_label 1;

done;

# Example: FD1 model for RNA off-target

mkdir rnaofftarget_eval_summary;

for i in fd1_model_eval_result/*_dnabert2_test_pred.csv; do

 docker run --platform=linux/amd64 --rm \

 -v `pwd`:/DATA -w /DATA \

 -i kazukinakamae/dnabert-eval:amd64 \

 python DNABERT_2_helper/evaluate_predictions_csv.py \

 --gold_file "fd1_model_eval_result/$(basename "$i")" \

 --pred_file "fd1_model_eval_result/$(basename "$i")" \

 --out_prefix rnaofftarget_eval_summary/fd1_model_eval_"$(basename "$i" .csv)" \

 --gold_label_column label \

 --pred_label_column pred_label \

 --id_column sample_id \

 --average macro \

 --pos_label 1;

done;

# Example: FD1 model for non-WCW RNA off-target

mkdir nonWCWrnaofftarget_eval_summary;

for i in fd1_model_eval_result/*_nonWCWrnaofftarget_test_dnabert2_test_pred.csv; do

 docker run --platform=linux/amd64 --rm \

 -v `pwd`:/DATA -w /DATA \

 -i kazukinakamae/dnabert-eval:amd64 \

 python DNABERT_2_helper/evaluate_predictions_csv.py \

 --gold_file "fd1_model_eval_result/$(basename "$i")" \

 --pred_file "fd1_model_eval_result/$(basename "$i")" \

 --out_prefix nonWCWrnaofftarget_eval_summary/fd1_model_eval_"$(basename "$i" .csv)" \

 --gold_label_column label \

 --pred_label_column pred_label \

 --id_column sample_id \

 --average macro \

 --pos_label 1;

done;

**8. Heatmap-based visualization**

Using *DNABERT_2_helper/merge_metrics_csvs.py*, aggregate all <prefix>_metrics.csv files within the specified directory and generate merged_metrics.csv. *DNABERT_2_helper/plot_heatmaps_from_merged.py* then uses merged_metrics.csv to render the heat maps for each performance metric. The baseline results can be explicitly specified using the “--baseline_model” option.

*Note: If replicate data are required, prepare separate directories for each replicate, named in the format rep<index> (e.g., promoter_eval_summary/rep1, promoter_eval_summary/rep2, ...).*

# Example: promoter classification

# Merge <prefix>_metrics.csv

docker run --platform=linux/amd64 --rm \

 -v `pwd`:/DATA -w /DATA \

 -i kazukinakamae/dnabert-eval:amd64 \

 python DNABERT_2_helper/merge_metrics_csvs.py \

 --input_glob "promoter_eval_summary/**/*_metrics.csv" --recursive \

 --rep_regex "(?:^|/|_)(?:rep|repeat|r)(?P<rep>\\d+)(?:/|_|$)" \

 --out_csv promoter_eval_summary/merged_metrics.csv;

# Create heatmaps

docker run --platform=linux/amd64 --rm \

 -v `pwd`:/DATA -w /DATA \

 -i kazukinakamae/dnabert-eval:amd64 \

 python DNABERT_2_helper/plot_heatmaps_from_merged.py \

 --input_csv promoter_eval_summary/merged_metrics.csv \

 --outdir promoter_eval_summary \

 --dpi 500 \

 --formats png,tiff,pdf \

 --agg mean \

 --baseline_model cnn_mpc_model \

 --draw_baseline_separator \

 --annotate;

# Example: CBE-specific RNA off-target prediction

# Merge <prefix>_metrics.csv

docker run --platform=linux/amd64 --rm \

 -v `pwd`:/DATA -w /DATA \

 -i kazukinakamae/dnabert-eval:amd64 \

 python DNABERT_2_helper/merge_metrics_csvs.py \

 --input_glob "rnaofftarget_eval_summary/**/*_metrics.csv" --recursive \

 --rep_regex "(?:^|/|_)(?:rep|repeat|r)(?P<rep>\\d+)(?:/|_|$)" \

 --out_csv rnaofftarget_eval_summary/merged_metrics.csv;

# Create heatmaps

docker run --platform=linux/amd64 --rm \

 -v `pwd`:/DATA -w /DATA \

 -i kazukinakamae/dnabert-eval:amd64 \

 python DNABERT_2_helper/plot_heatmaps_from_merged.py \

 --input_csv rnaofftarget_eval_summary/merged_metrics.csv \

 --outdir rnaofftarget_eval_summary \

 --dpi 500 \

 --formats png,tiff,pdf \

 --agg mean \

 --baseline_model NNN_motif \

 --baseline_model ACW_motif \

 --baseline_model WCW_motif \

 --draw_baseline_separator \

 --annotate;

# Example: CBE-specific RNA off-target prediction models in the non-WCW motif dataset

# Merge <prefix>_metrics.csv

docker run --platform=linux/amd64 --rm \

 -v `pwd`:/DATA -w /DATA \

 -i kazukinakamae/dnabert-eval:amd64 \

 python DNABERT_2_helper/merge_metrics_csvs.py \

 --input_glob "nonWCWrnaofftarget_eval_summary/**/*_metrics.csv" --recursive \

 --rep_regex "(?:^|/|_)(?:rep|repeat|r)(?P<rep>\\d+)(?:/|_|$)" \

 --out_csv nonWCWrnaofftarget_eval_summary/merged_metrics.csv;

# Create heatmaps

docker run --platform=linux/amd64 --rm \

 -v `pwd`:/DATA -w /DATA \

 -i kazukinakamae/dnabert-eval:amd64 \

 python DNABERT_2_helper/plot_heatmaps_from_merged.py \

 --input_csv nonWCWrnaofftarget_eval_summary/merged_metrics.csv \

 --outdir nonWCWrnaofftarget_eval_summary \

 --dpi 500 \

 --formats png,tiff,pdf \

 --agg mean \

 --baseline_model WCW_motif \

 --draw_baseline_separator \

 --annotate;

**Result interpretation**

**Figure 1.**
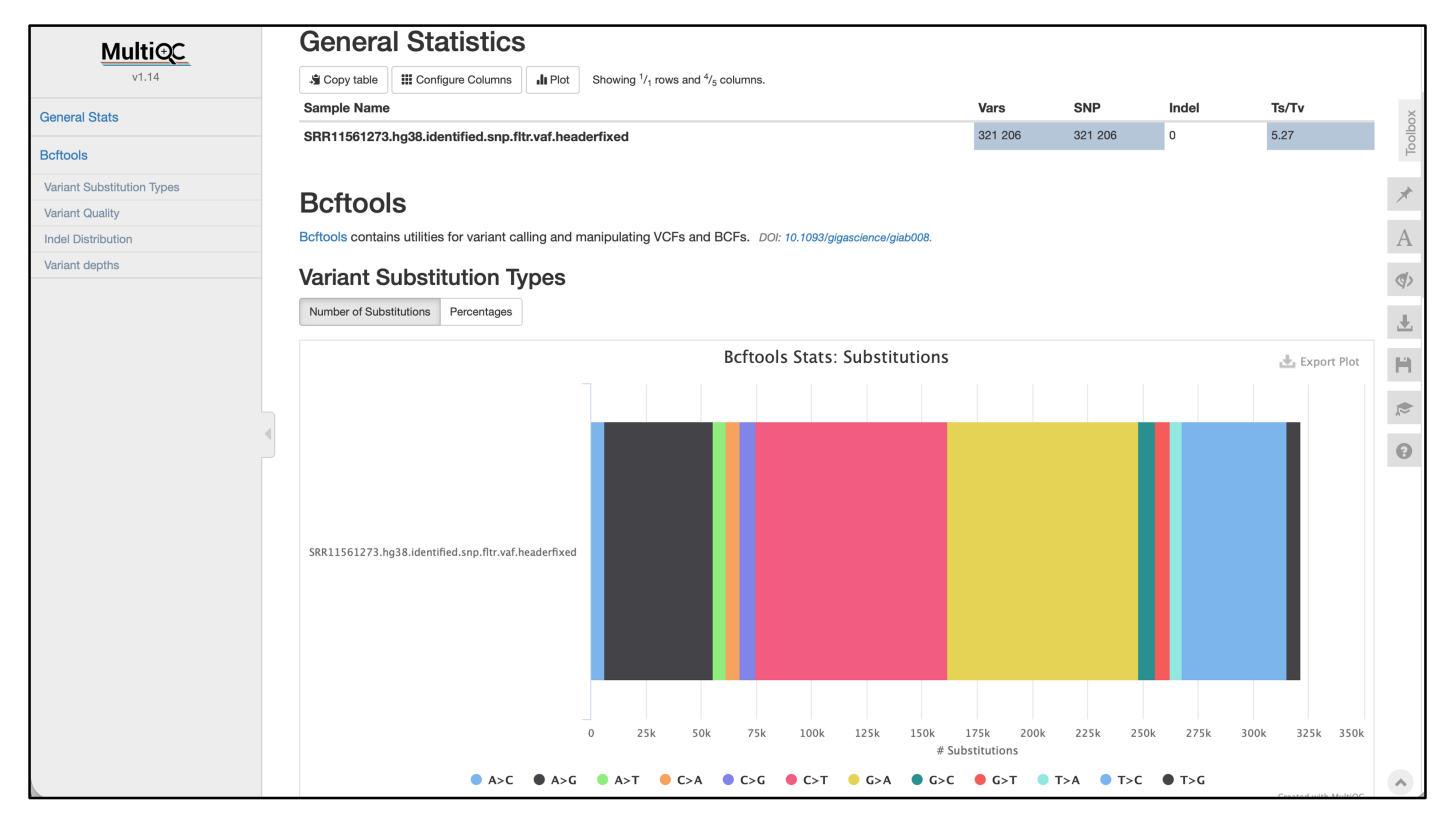
MultiQC report for RNA variant detection by PiCTURE. The report summarizes the detected variants and presents the corresponding statistics and bar plots. The data is saved as *figure/multiqc_report.html*.

**Figure 2.**
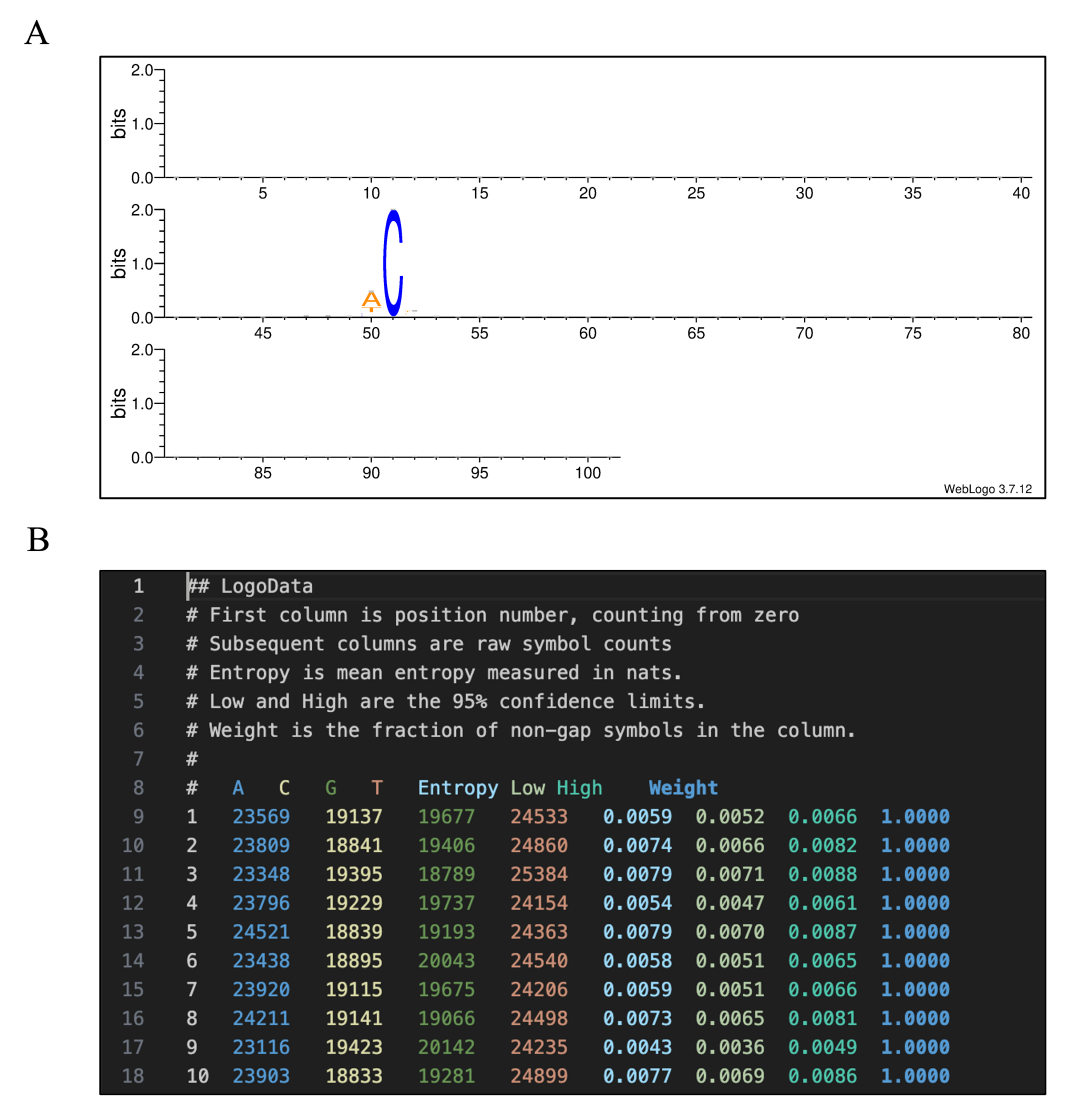
Motif analysis results from PiCTURE. (A) Sequence logo [35] of ±50 bp flanking regions around the detected variant sites. Each position has a stack of DNA letters (A, T, C, G). The height of the entire stack represents the frequency of that position. A high stack means high frequency, while a low stack indicates low frequency. Within a stack, the most frequent bases are shown on top as the largest letter, while less frequent ones appear smaller below. The data is saved as SRR11561273.hg38.identified.snp.fltr.CtoT.fa.png. (B) Position-wise nucleotide occurrence counts within the ±50 bp flanking regions, together with information entropy, the 95% confidence interval, and positional weight information. The data is saved as SRR11561273.hg38.identified.snp.fltr.CtoT.fa.txt.

The PiCTURE report files generated in section A1.5 include data for all detected variants (the “all” directory) as well as data partitioned by the variant-frequency threshold specified in motif_estimation.sh (the “each” directory). Each directory contains a MultiQC-based visualization report of variant statistics (the “figure” directory) [26], motif analysis results (the “motif” directory), sequence sets covering detected variant sites and their ±50 bp flanking regions (the “sequence” directory), and variant calling results (the “vcf” directory). The proportion of detected variants was inspected in a web browser using *figure/multiqc_report.html* (Figure 1). This data provides an overview of the number and types of substitution variants. In particular, for CBE-treated samples, it is advisable to qualitatively confirm that C > T or G > A substitutions are more prevalent than other substitution types. If other substitution types appear to be dominant, it may indicate that the analysis samples were mixed up or that the observed variants arise from phenomena unrelated to CBE-mediated off-target effects. Because it is unlikely for fewer than 1,000 variants to be detected in this analysis framework, it is recommended to re-examine the sequence data used for the analysis in this case. The motif directory contained sequence logos and nucleotide count information derived from the sequences in which the C > T substitutions were detected (Figure 2A, B). Because only C > T substitution variants were extracted in Figure 2A, it is expected that the motif at position 51 is C. The bases of interest are therefore the surrounding nucleotide motifs, which can be interpreted as the flanking sequence context frequently observed when C > T substitutions occur. In Figure 2B, the actual frequency values can be examined, and if replicate data are available, the observed patterns can also be evaluated using statistical hypothesis testing. Moreover, these variant statistics can be examined for each variant frequency threshold, enabling comparisons of motif differences between low-(<0.8) and high-frequency (>0.8) variants.

**Figure 3.**
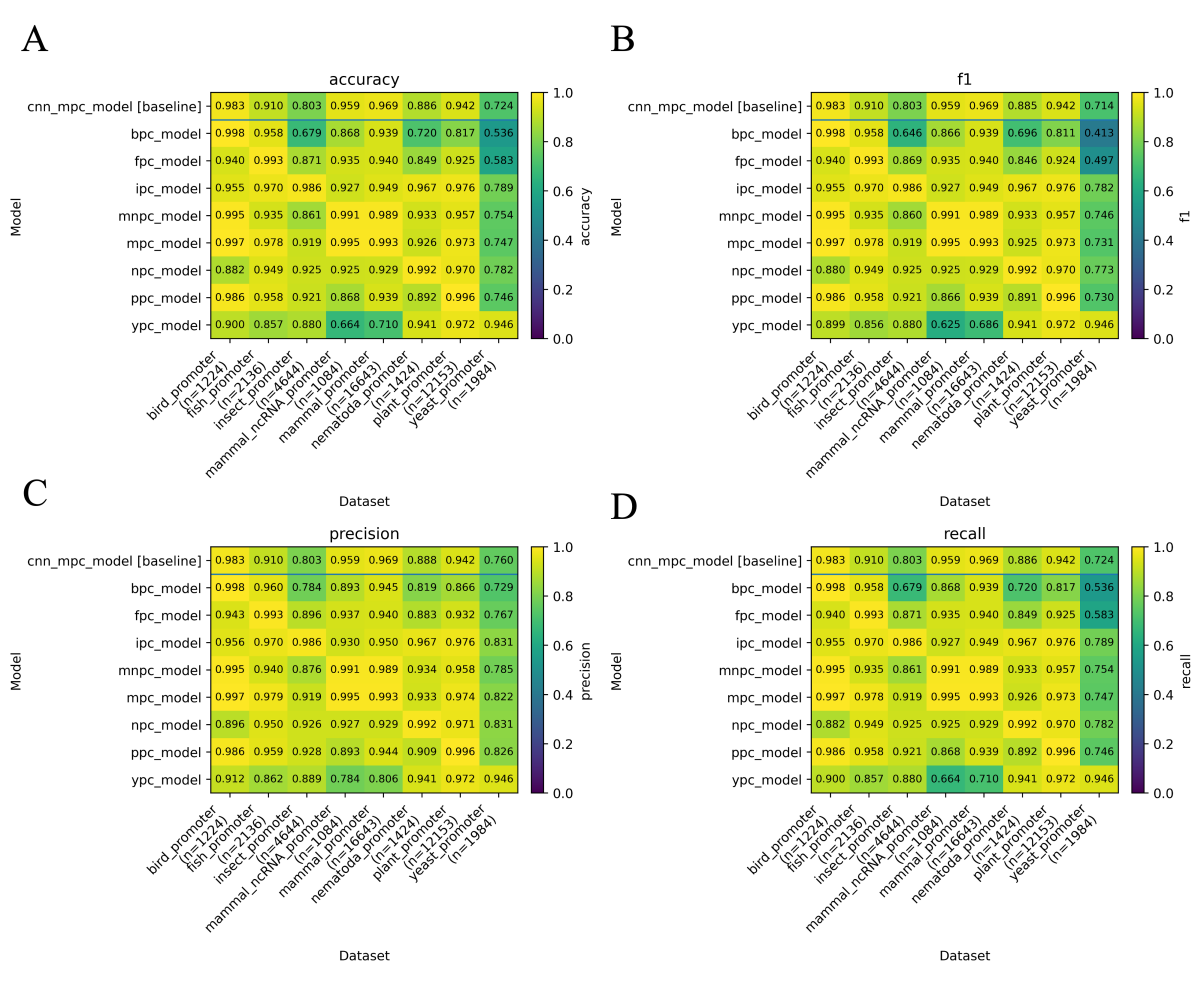
Heatmaps of performance metrics for the promoter prediction models. Heatmaps summarizing (A) accuracy, (B) F1 score, (C) precision, and (D) recall for each promoter prediction model [bird: bpc_model; fish: fpc_model; insect: ipc_model; mammal (ncRNA): mnpc_model; mammal: mpc_model; nematoda: npc_model; plant: ppc_model; and yeast: ypc_model] evaluated on the corresponding test datasets [bird: bird_promoter; fish: fish_promoter; insect: insect_promoter; mammal (ncRNA): mammal_ncRNA_promoter; mammal: mammal_promoter; nematoda: nematoda_promoter; plant: plant_promoter; and yeast: yeast_promoter]. The cnn_mpc_model denotes the 3-layer one-hot CNN baseline. The n indicates sample size. The data is saved in *promoter_eval_summary*.

The performance metrics of the promoter classification models built using DNABERT-2 are summarized using the heat maps generated in section C8 (Figure 3A, B). Across models and datasets corresponding to each taxonomic group, the DNABERT-2 models consistently achieved higher performance than the 3-layer one-hot CNN baseline. In general, the highest prediction accuracy is expected from models trained on data from the same species as the evaluation dataset. In this example, the baseline model was constructed using mammalian data; therefore, it is not a major concern if models trained on data from other species perform slightly worse than the baseline. However, if any evaluation metric falls below 0.5, it is likely that the training has failed, and the dataset should be carefully re-examined.

**Figure 4.**
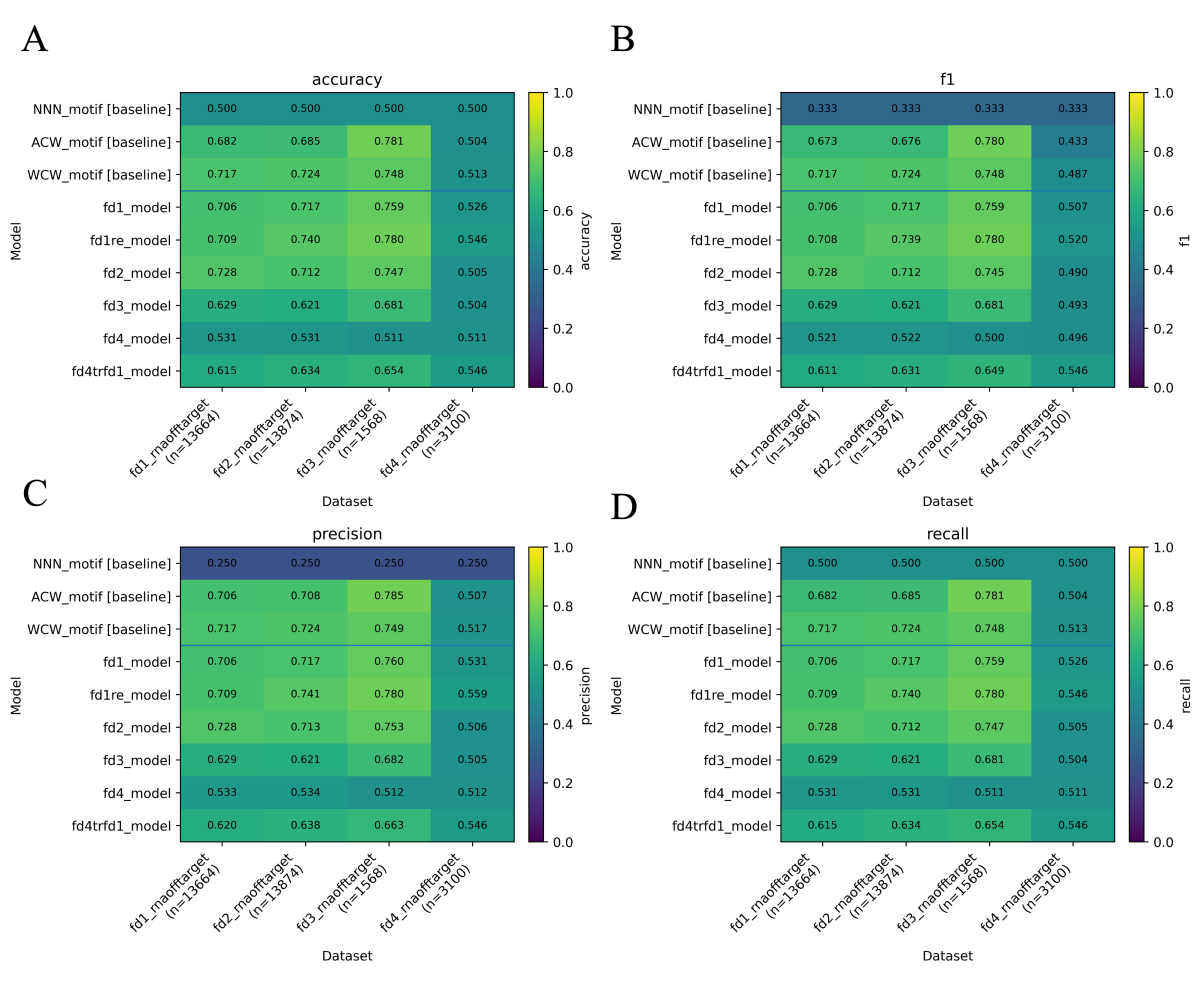
Heatmaps of performance metrics for the cytosine base editor (CBE)-specific RNA off-target prediction models. Heatmaps summarizing (A) accuracy, (B) F1 score, (C) precision, and (D) recall for each CBE-specific RNA off-target prediction model (fd1_model, fd1re_model, fd2_model, fd3_model, fd4_model, fd4trfd1_model) evaluated on the corresponding test datasets (BE4-rAPOBEC1/single-genotyping: fd1_rnaofftarget, BE4-rAPOBEC1/joint-genotyping:fd2_rnaofftarget, BE4-rAPOBEC1/intersection:fd3_rnaofftarget, and BE4-RrA3F/single-genotyping: fd4_rnaofftarget). The NNN_motif, ACW motif, and WCW motif denote the motif-based baseline. The n indicates sample size. The data is saved in *rnaofftarget_eval_summary*.

The performance metrics of the CBE-specific RNA off-target models built using DNABERT-2 are summarized using the heat maps generated in section C8 (Figure 4). The baseline evaluation based on the NNN motif represents the theoretical accuracy achievable by a model that cannot predict negative cases. Therefore, performance approaching metrics value from NNN motif classification indicates a failure in the model construction itself. In contrast, the baseline evaluations based on the ACW and WCW motifs reflect prediction accuracies derived from criteria known to be effective. The metric values, which are lower than ACW and WCW motif-based evaluations, do not indicate a failure in model construction. Compared to the motif-based baseline, the fd1 model, which was trained on merged single-genotyping data derived from BE4-rAPOBEC1–treated samples, outperformed the ACW motif-based rule on the hold-out dataset (fd1_rnaofftarget). In contrast, its performance was comparable to that of the WCW motif-based rule, with no significant differences observed. The f1re model, which was further trained on the same dataset, exhibited an overall improved performance across the metrics. The fd2 model, trained on joint genotyping data derived from BE4-rAPOBEC1–treated samples, achieved a performance comparable to that of the fd1 model. In comparison, the fd3 (trained on variants shared between two samples) and fd4 models (trained on variants derived from BE4-RrA3F–treated samples) performed poorly, even relative to the baseline. One possible explanation is that their training datasets contain fewer than one-third of the samples used for fd1 and fd2, which likely limits the generalization performance.

Notably, the fd4trfd1 model, which was obtained by continuing the training of the fd1 model using data derived from the BE4-RrA3F–treated samples, achieved the best performance on the BE4-RrA3F hold-out dataset (fd4_rnaofftarget). This result suggests that when constructing models for a target condition with limited sample sizes, it can be effective to first train a model on a larger dataset obtained under similar conditions and subsequently fine-tune the model on the target-condition dataset.

**Figure 5.**
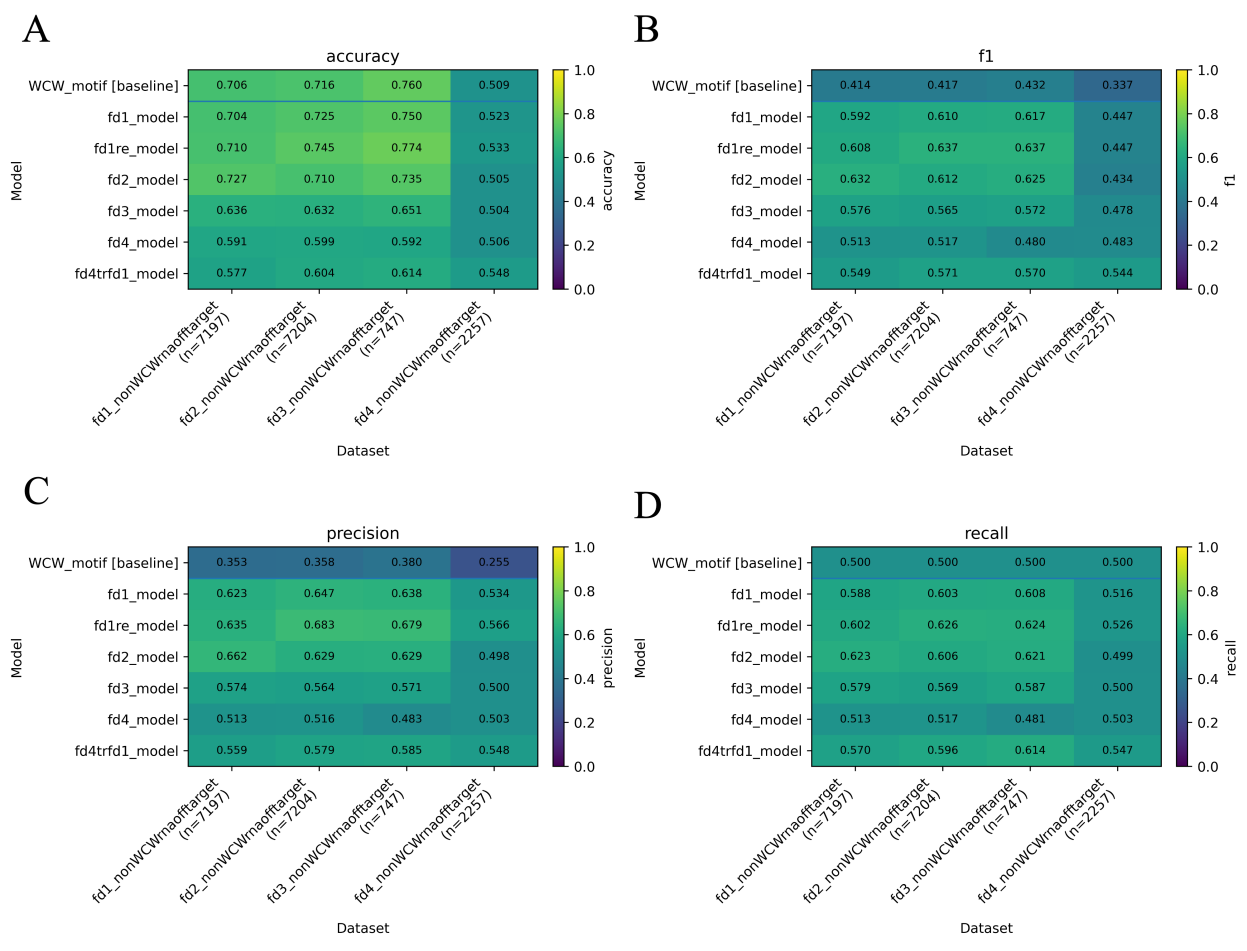
Heatmaps of performance metrics for the cytosine base editor (CBE)-specific RNA off-target prediction models in the non-WCW motif dataset. Heatmaps summarizing (A) accuracy, (B) F1 score, (C) precision, and (D) recall for each CBE-specific RNA off-target prediction model (fd1_model, fd1re_model, fd2_model, fd3_model, fd4_model, fd4trfd1_model) evaluated on the corresponding non-WCW motif datasets from test datasets (BE4-rAPOBEC1/single-genotyping: fd1_rnaofftarget, BE4-rAPOBEC1/joint-genotyping:fd2_rnaofftarget, BE4-rAPOBEC1/intersection:fd3_rnaofftarget, and BE4-RrA3F/single-genotyping: fd4_rnaofftarget). The WCW motif denotes the motif-based baseline. The n indicates sample size. The data is saved in *nonWCWrnaofftarget_eval_summary*.

Furthermore, when evaluating the model performance on datasets lacking the WCW motif, the f1re model yielded the best overall accuracy among the BE4-rAPOBEC1–derived datasets (fd1_rnaofftarget, fd2_rnaofftarget, and fd3_rnaofftarget) (Figure 5). In the dataset shown in Figure 5, the WCW motif–based evaluation represents the theoretical accuracy achieved by a model that cannot predict positive cases. Therefore, achieving an accuracy higher than the WCW motif–based baseline indicates the model’s ability to detect RNA off-target events occurring at non-WCW motifs. The result of f1re model may reflect enhanced sequence recognition, which is less dependent on the presence of motifs because of the additional training.

As described above, we compared multiple models, including baseline methods. However, without sufficiently large training datasets and appropriate hyperparameter tuning, a trained model may underperform the baseline. Furthermore, because pretrained models have already been trained on large-scale DNA sequence data, it is difficult to predict in advance how much task-specific data will be sufficient for effective fine-tuning in a given task. Therefore, we strongly recommend explicitly defining the baseline methods in all evaluations. If a specific metric is of primary interest, the “--metric_name” option in *DNABERT_2_helper/select_best_run.py* (section B2.4) can be used to select that metric instead of “eval_loss” when choosing the best run. In addition, a grid search followed by empirically guided manual adjustment can be effective in improving performance. A prior report showed that manual curation of hyperparameters can yield models that outperform WCW motif-based rules. In practice, adjusting the learning rate tends to be particularly impactful during such manual curation.

## Validation of protocol

This protocol has been validated as shown in the Result interpretation section. We performed a similar analysis in a published study on CBE-induced RNA off-targets, where DNABERT-2 models fine-tuned using the described data preparation and training steps achieved improved accuracy, precision, recall, and F1 scores compared to motif-based baselines (see original research article, figures, and tables corresponding to the STL and SNL models [13]).

In addition, we applied the same workflow to plant promoter prediction using EPDnew-derived promoter sequences and dinucleotide shuffling sequences. The fine-tuned DNABERT-2 model achieved robust discrimination between promoter and non-promoter sequences (e.g., F1 score ≈ 0.98–0.99 on held-out test sets) in the section of Result interpretation, demonstrating the generality of the protocol beyond RNA off-target tasks.

Together, these applications demonstrate that the protocol can be reproduced across independent datasets and adapted for distinct genomic tasks, providing confidence that other users can obtain reliable results by following the described steps.

## General notes and troubleshooting

**General notes**

1. Defining positive and negative datasets: developers should carefully consider the intended use case when defining what constitutes the positive and negative datasets. For example, in the promoter prediction task described here, the promoter sequences in the genome were treated as positive data, whereas their sequence-randomized counterparts were used as negative data. Such randomized sequences are likely to differ substantially from genomic sequences that carry diverse biological functions and can therefore be regarded as non-genomic. Consequently, when applying the model to real genomic sequences, even non-promoter regions may still appear “genome-like,” which could bias predictions toward the positive class.

In contrast, for applications such as evaluating artificially designed promoter sequences, the promoter prediction model may still be practically useful for sequence-level assessments. Similarly, in the CBE-specific RNA off-target detection model, the positive class corresponds to CBE-specific C-to-U (T) substitutions, whereas the negative class corresponds to CBE-non-specific C-to-U (T) substitutions. Therefore, a negative prediction using this model does not imply that mutations are unlikely to occur. This model is expected to be effective in distinguishing regions in which the mutation risk increases, specifically upon CBE transfection, from regions in which the risk remains unchanged.

Overall, dataset selection should be guided by a deep understanding of biological or experimental phenomena that cannot be represented by the chosen labeling scheme.

2. Addressing the label imbalance when using alternative data sources: When preparing a fine-tuned model using approaches other than this protocol, particular attention should be paid to potential label biases. Depending on the biological phenomenon of interest, the prepared dataset may exhibit substantial class imbalance, which can lead to inappropriate training behavior and misleading performance evaluation. We recommend balancing the class labels prior to fine-tuning.

**Troubleshooting**

**Problem 1:** PiCTURE preparation.sh completes, but reference genome files are missing or very small.

Possible cause: The downloaded file may be an error page (XML/HTML), or the download may have been interrupted.

Solution: We recommend checking the first line of Homo_sapiens_assembly38.fasta (should start with >). Re-run preparation.sh or manually re-download the GATK resource bundle for hg38 using the following link:

https://gatk.broadinstitute.org/hc/en-us/articles/360035890811-Resource-bundle or https://doi.org/10.6084/m9.figshare.31742074.

**Problem 2:** The PiCTURE pipeline does not complete, or the output directories are empty after finishing *run.sh*.

Possible cause: PiCTURE is memory-intensive and can require a prolonged runtime. The process may have terminated because it exhausted the available memory and aborted, or because the session was disconnected.

Solution: We recommend the use of a server equipped with at least 120 GB of RAM. In addition, to ensure that the processing continues even if the session is disconnected, it is preferable to run the pipeline in the background.

**Problem 3:** Unexpectedly small number of extracted sequences from the PiCTURE output.

Possible cause: Variant filtering thresholds were too strict, or the input VCF is empty due to FASTQ/VCF mismatch and wrong sample grouping.

Solution: Inspect the identified VCF (check that it contains variants) and confirm sample/group labels using the *less* command. Try relaxing filters or verifying that mapping produced non-empty BAM files using the *samtools flagstat* command. If the response shows “*0 + 0 in total*”, the BAM file is empty.

docker run --rm --platform=linux/amd64 -v "$PWD:/input_data" staphb/samtools:1.22.1 \

 bash -lc "samtools flagstat /input_data/XXX.bam";

**Problem 4:** Fine-tuning cannot be executed with *train.py*.

Possible cause: The GPU server environment is likely not configured correctly.

Solution: After creating a backup, it is recommended to reinitialize the server and repeat sections B1–2.

**Problem 5:** Inferences using the DNABERT-2 model cannot be performed on a client PC.

Possible cause: The Docker environment may not have been set up correctly.

Solution: The Docker image is run on a Linux/amd64 platform. Rosetta 2 must be installed when using an Apple Silicon Mac. In addition, the inference may fail if Triton is used. Although Triton is excluded from the provided Docker image, we recommend verifying this by running the following command and confirming that the output is “None.”

docker run --rm --platform=linux/amd64 kazukinakamae/dnabert-eval:amd64 \

 bash -lc 'python -c "import importlib; print(importlib.util.find_spec(\"triton\"))"';

If Triton is detected, modify DNABERT_2_helper/Dockerfile to match your local environment.

**Problem 6:** The fine-tuned model does not outperform the baseline.

Possible cause: Insufficient hyperparameter tuning and/or insufficient training data.

Solution: Although this protocol demonstrates optimization using a relatively simple grid search, it is preferable to evaluate a larger number of hyperparameter combinations for practical applications. In particular, the learning rate has a substantial impact on the performance; therefore, a broad range of learning-rate candidates should be included. After running an initial grid over a reasonable range, it can be effective to visually inspect the performance trends and either manually select parameters or conduct a second, narrower grid search focused on promising regions. Implementing alternative approaches such as Bayesian optimization [40] and Asynchronous Successive Halving Algorithm (ASHA) [41] is also an effective option. However, if the evaluation metric remains below 0.5, limited data are likely, and dataset expansion should be considered. Additionally, it is advisable to verify whether the labels are imbalanced or whether the dataset construction inadvertently favors the baseline method. If such issues are suspected, regeneration of a fair dataset under the revised conditions should be considered.
